# Carbon dots for virus detection and therapy

**DOI:** 10.1007/s00604-021-05076-6

**Published:** 2021-11-25

**Authors:** Jan Belza, Ariana Opletalová, Kateřina Poláková

**Affiliations:** 1 Regional Centre of Advanced Technologies and Materials, Czech Advanced Technology and Research Institute (CATRIN), Palacký University Olomouc, Šlechtitelů 27, 779 00 Olomouc, Czech Republic; 2 Department of Physical Chemistry, Faculty of Science, Palacký University Olomouc, 17. listopadu 1192/12, 771 00 Olomouc, Czech Republic

**Keywords:** Antiviral, Biosensors, Carbon dots, Coronavirus, COVID-19, Functionalization of carbon dots, Virus detection

## Abstract

Recent experience with the COVID-19 pandemic should be a lesson learnt with respect to the effort we have to invest in the development of new strategies for the treatment of viral diseases, along with their cheap, easy, sensitive, and selective detection. Since we live in a globalized world where just hours can play a crucial role in the spread of a virus, its detection must be as quick as possible. Thanks to their chemical stability, photostability, and superior biocompatibility, carbon dots are a kind of nanomaterial showing great potential in both the detection of various virus strains and a broad-spectrum antiviral therapy. The biosensing and antiviral properties of carbon dots can be tuned by the selection of synthesis precursors as well as by easy post-synthetic functionalization. In this review, we will first summarize current options of virus detection utilizing carbon dots by either electrochemical or optical biosensing approaches. Secondly, we will cover and share the up-to-date knowledge of carbon dots’ antiviral properties, which showed promising activity against various types of viruses including SARS-CoV-2. The mechanisms of their antiviral actions will be further adressed as well. Finally, we will discuss the advantages and distadvantages of the use of carbon dots in the tangled battle against viral infections in order to provide valuable informations for further research and development of new virus biosensors and antiviral therapeutics.

**Graphical abstract:**

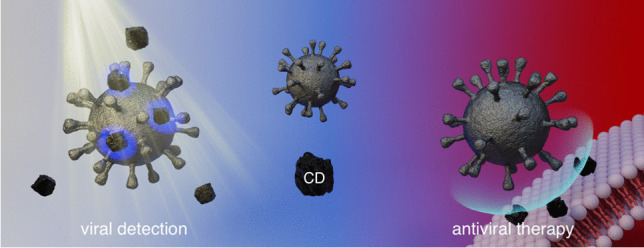

## Introduction

In 1977, biologist Peter Medawar wrote that virus is “simply a piece of bad news wrapped up in protein” [1]. The question is whether a virus is a living entity or not. If not, is it possible at all to kill something that is dead? We have to take this into account when facing viral infections. Since viruses are very small particles that can be seen only by advanced microscopic techniques, they are not easy to detect. Moreover, as they hijack the metabolism of host cells, it is hard to destroy them. The ultimate task for a virus is the duplication of its genetic material and controlled expression of the encoded information. To achieve this, all viruses have to synthesize their messenger ribonucleic acid (mRNA) and translate it via the metabolic apparatus of the host cell. According to the type of the genetic material that the virus contains and the pathway of mRNA synthesis, viruses can be divided into seven groups following the so-called Baltimore classification. The type of nucleic acid that the virus contains can be single-stranded DNA (ssDNA), sense ssRNA, antisense ssRNA, double-stranded DNA (dsDNA), and dsRNA [2]. Viruses are responsible for about one-third of deaths from infectious diseases [3]. They are the cause of many human diseases ranging from common cold [4] to infections as severe as rabies [5] or Ebola virus disease [6]. Clearly, vaccination is the best defense against viral infections; however, it may take a long time until the process of its development and approval is completed. In extreme cases such as the human immunodeficiency virus (HIV), vaccine has not still been developed, nearly four decades since the onset [7]. As a consequence, we must draw up innovative strategies to effectively face emerging viral infections, especially those which we do not have vaccine against. In 2021, more than a hundred years after the outbreak of the Spanish flu in 1918, we are facing a new and serious threat called COVID-19, a disease caused by severe acute respiratory syndrome coronavirus 2 (SARS-CoV-2) [8]. Therefore, it is now crucial to invest hard effort in the development of new techniques for virus detection and therapy. In this review, we will focus on carbon dots (CDs)—nanoscale materials—which, thanks to their unique properties, could provide an opportunity to develop a flexible, broad range antiviral therapeutics and high-sensitivity virus detection platforms [9, 10].

## Carbon dot**s**

In the twenty-first century, considerable efforts have been devoted to the research and development of new nanomaterials based on a wide variety of carbon allotropes such as graphite, graphene, or graphene oxide (2D); carbon nanotubes, nanofibers, nanoribbons (1D); and zero-dimensional fullerenes, nanodiamonds, and CDs. These materials have unique physicochemical properties and therefore offer a broad application potential, for example, in photonics, electronics, biomedicine, or renewable energy [11–16].

Among carbon nanomaterials, CDs with their size usually below 10 nm represent an emerging class of highly fluorescent 0D nanoparticles. The invention and the subsequent intensive research into CDs started more than 17 years ago [17] when these first fluorescent 0D nanoparticles were prepared from arc-discharge soot. Thanks to their small size and special structure, CDs display unique fluorescence properties and can therefore emit light with high photoluminescent quantum yield (QY) after appropriate irradiation. QY is described as the ratio between the numbers of emitted and absorbed photons. It has been shown that the fluorescence of CDs is highly stable and resistant to photobleaching and photoblinking, which is advantageous, especially when compared to commonly used organic dyes or polymer dots [18, 19]. Moreover, their chemical inertness, hydrophilicity, and excellent biocompatibility make them more favorable for applications in biological and biomedical fields than heavy metal-containing semiconductor QDs [20–26].

**Fig. 1:**
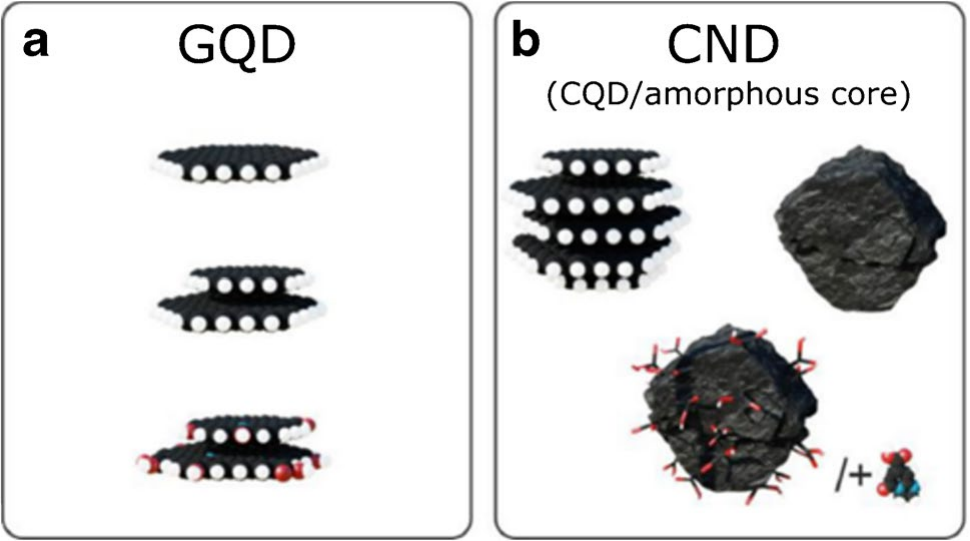
Scheme of CDs: **a** GQDs — graphene quantum dots and **b** CNDs — carbon nanodots with either graphitic (CQDs) or amorphous core. *Reprinted from* [27]*, Page No. 2, Copyright 2021, with permission from Elsevier*

Mainly two types of CDs are used in bioapplications thanks to their advanced fluorescent properties, namely graphene quantum dots (GQDs) and carbon quantum dots (CDs) [20]. Structurally, they are slightly different, which is mainly due to the synthetic procedure by which they were prepared. Consequently, also their physicochemical properties differ considerably [27]. While GQDs are only one- or a few-layered graphene nanosheets prepared usually by the “top-down” synthesis, CDs, on the other hand, are quasi-spherical globular particles with a graphitic core synthesized by the “bottom-up” approach. Both of them, however, reveal sp^2^ hybridization with high oxygen content (Fig. 1) [22, 27]. Regarding bioapplications, appropriate functional groups are often required for further derivatization and the binding of biological active compounds such as antibodies, drugs, proteins, or nucleic acids. Moreover, thanks to the ultra-small size of CDs that is usually below 10 nm, they have extraordinarily high specific surface area, on which the exceptionally large amount of these biological substances can be easily bound. The majority of CDs have on their surfaces various functional groups such as carboxyl, amino, sulfur, or epoxy moieties, which can come directly from the synthesis or post-synthetic functionalization by appropriate polymers with the desired functionalities [28].

**Fig. 2:**
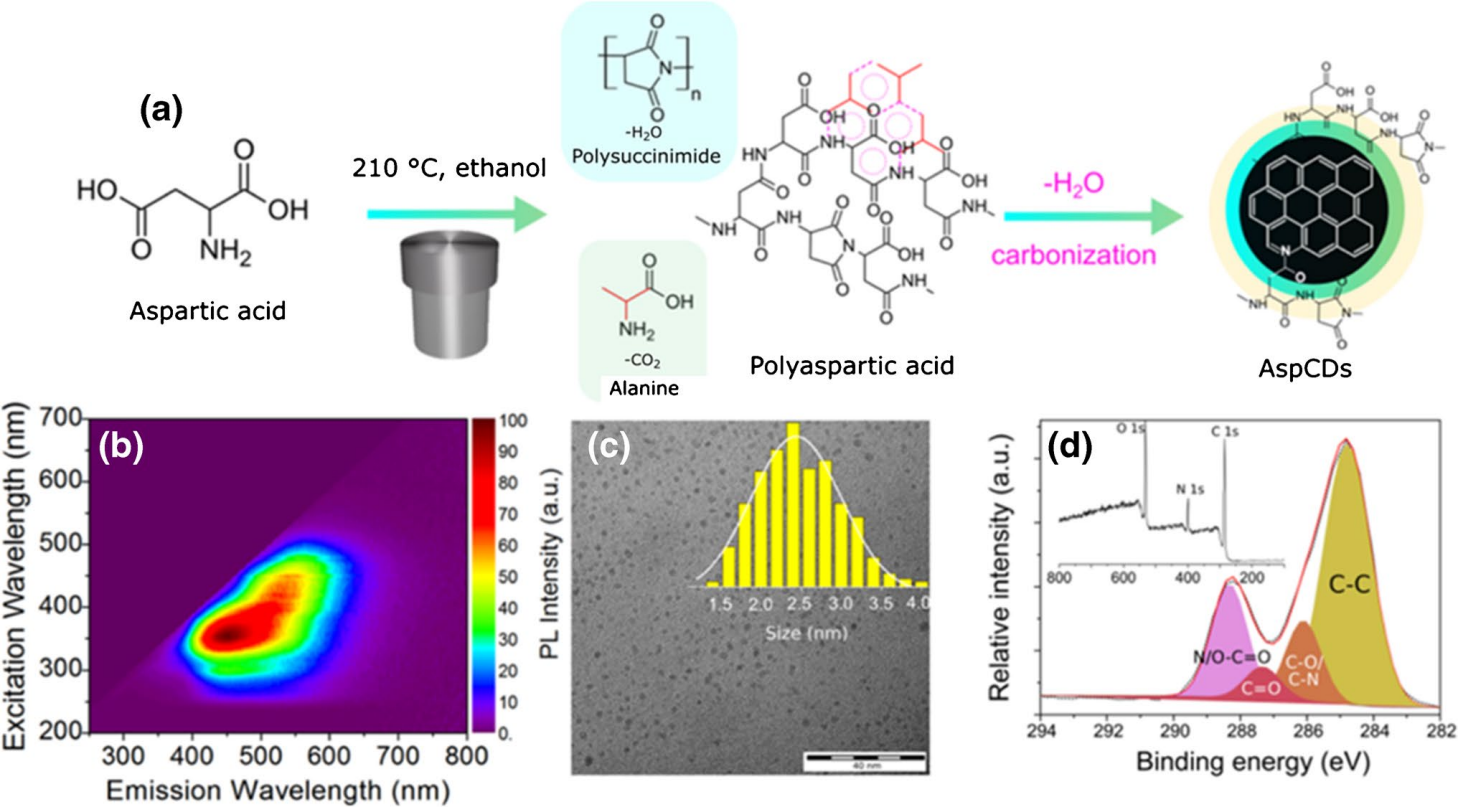
**a** Reaction scheme of AspCDs preparation; **b** fluorescent excitation–emission map for prepared AspCDs in water; **c** TEM image of AspCDs with size distribution in the inset, and the scale bar is 40 nm; and **d** XPS survey spectrum (inset) and the high-resolution C 1s XPS spectrum of AspCDs. *Reprinted with permission from* [62]*, Page No. 9944. Copyright 2020 American Chemical Society*

Top-down syntheses usually start with graphene, graphite, or other carbon precursors such as fullerenes or nanotubes, and with various harsh physical or chemical treatments such as oxygen plasma treatment, laser ablation, electrochemical oxidation, or chemically oxidative cutting ablation; the size of the produced CDs is finally reduced down to 25 nm and less [20, 27]. The surface of such a CDs is usually passivated to reach higher photoluminescence (PL) QY [29]. During bottom-up syntheses, the controlled thermal oxidation of organic precursors in autoclave or microwave enables to prepare CDs with defined sizes, surfaces, and functional groups, which lends them their high fluorescence efficiency and high QY without the need for final surface passivation [27]. In this way, CDs can be prepared by carbonization (polycondensation reaction) of any organic substance. The reaction scheme of CDs synthesized simply from aspartic acid and their corresponding optical, size, and structural properties are depicted in Fig. 2. Interestingly, CDs have been already prepared even through green synthetic ways utilizing various food substances such as green tea [30], pomelo [31] and watermelon peels [32], orange peel waste [33], overcooked barbecue [34], and chicken eggs [35]. In general, both types of the syntheses (top-down and bottom-up) are cost-effective, sustainable, and robust which leads to novel 0D fluorescent CDs with extraordinary properties.

**Fig. 3:**
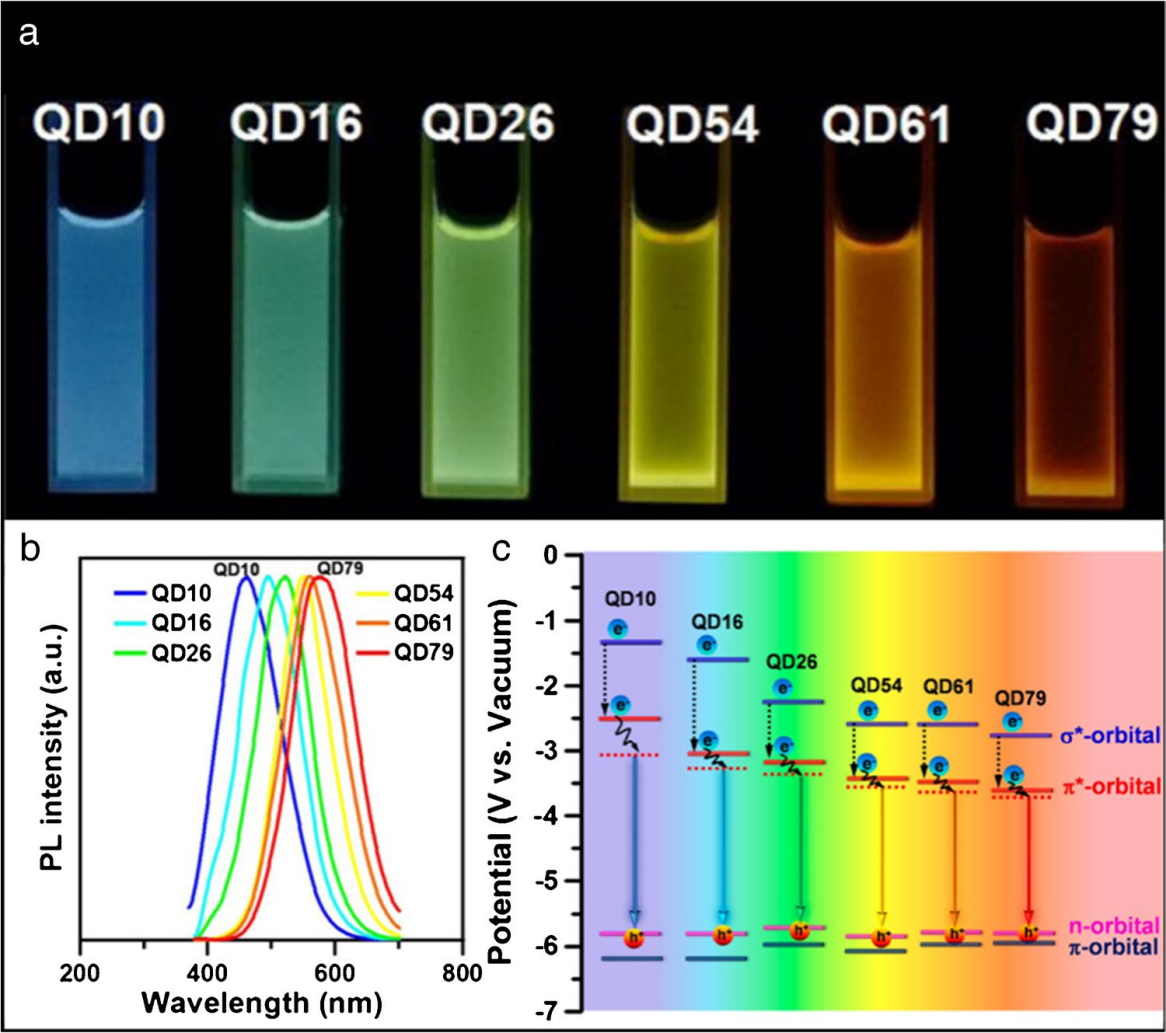
Size-dependent excitation/emission of CDs. **a** Images of GQDs of different sizes under UV lamp irradiation (QD10 means 1.0 nm, etc.), **b** normalized PL spectra excited at 350 nm that indicates size-dependent emission wavelength, and **c** schematic energy level diagram of GQDs. *Reprinted with permission from* [36]*. Copyright 2016 American Chemical Society*

The mechanism behind the origin of fluorescence in CDs is, however, still under extensive experimental and also theoretical research. As it is known quite precisely, the main emission peak in a semiconductor quantum dot is given by the size and shape of the core. Also in the case of CDs, the maximum emission and final color of the emitted light could be driven by the graphitic core size [20, 27, 29]. Generally, the creation of fluorescence is assigned to the radiative recombination of electrons and holes trapped on the surface of CDs. The smaller particles show emission in shorter wavelengths (blue, green). With the increasing size of the particle core, the fluorescence peak is shifted to the longer wavelengths between orange and near-infrared (NIR) region (Fig. 3a) [36]. Another possible and feasible way to obtain CDs with the defined color of emission from blue to red region of wavelength is by column chromatography (Fig. 3b) [37]. It was observed that the crucial factor in changing the color of the PL emission was the surface states of the CDs. The majority of the CDs show excitation-dependent broad emission usually in the middle of the visible spectra. However, in some types of syntheses, fluorescence is independent of excitation [38].

More importantly, wavelength emission and QY efficiency in CDs can be tuned by a dopant (usually nitrogen and sulfur) in the graphitic structure (Fig. 3c) and also by the additional surface functional groups (-CHO, =O, -COOH, -OH, -CONR_2_, or -NH_2_). It has been already reported that the presence of carboxylic or amino functional groups causes a decrease in the bandgap in the structure of CDs, leading to the shift in the fluorescence to higher wavelengths (red, NIR) [39]. The same red-shifted fluorescence was observed for CDs containing a higher amount of graphitic nitrogen in the structure [40]. Moreover, the PL profile is considerably dependent also on the pH or the solvent [41–44].

Even though there are a number of studies on CDs, some challenges still need to be tackled, including the enhancement of QY and preparation of CDs having emission wavelengths in NIR-I or NIR-II biowindows, accompanied with further theoretical studies dealing with the fundamental mechanisms of photoluminescence [27].

As we have recently shown, CDs are already used in a number of areas including optical applications (sensors [45], anticounterfeiting [46]), energy applications (light-emitting diodes, photovoltaics, supercapacitors), and catalytic applications [47, 48]. Moreover, CDs have attracted a tremendous interest in biomedicine, particularly in bioimaging (one-photon and two-photon imaging, live cell imaging, and tracking [49–53] and microbial [54] and plant tissue imaging), as well as in therapeutic applications including phototherapy, photodynamic therapy, and drug or gene delivery [20]. A newly studied application of CDs is their use for the detection of viruses; moreover, their interesting antiviral properties are being explored as well. This current review will be dedicated directly to these last two emerging applications of CDs.

## Biocompatibility of carbon dots

Although the prospects for the use of CDs in nanomedicine are broad, the potential risks arising from interactions between CDs and biological systems must also be considered [50, 55, 56]. We have already published several papers focusing on the biological use and cytotoxicity of different types of carbon dots [46, 53, 57]. In general, the toxicity is either determined by the chemical nature of the quantum dot core (Cd vs C) or, if we only talk about carbon dots, the potential toxicity is mainly determined by the type of surface modification (e.g., PEI vs PEG). Therefore, CDs show first significant toxic effects at concentrations typically in the order of hundreds of μg mL^−1^, which is at least ten times higher than for semiconductor quantum dots and several times higher than the concentrations needed for potential biological applications (10–100 μg mL^−1^). In addition, several studies suggest that CDs cause lower or similar cytotoxic effects as polymers used as passivating agents (PEI, PEG, etc.) [58, 59]. Moreover, we have recently studied carbon dots very precisely. Genome-wide mRNA and miRNA expression (RNAseq) as well as gene-specific DNA methylation changes have been analyzed. Although some changes at the genomic level (deregulation of processes and pathways) were detected, the overall decrease in viability of the studied cells started from 100 μg mL^−1^ for positively charged CDs and for negatively charged CDs, the viability reached more than 90% even at a concentration of 500 μg mL^−1^ [60]. These results are similar to, for example, iron oxide nanoparticles, which are considered biocompatible nanoparticles.

The most important factor for the potential use of fluorescent quantum dots is whether they will be used in vivo or ex vivo as part of biological immunoassays. The first systematic study of CDs in vivo conducted by Tao et al [59] concluded that there were no obvious side effects compared to the control group. CDs were rapidly excreted from the body in the urine. Completely new results from the work of Chung et al. [61] showed that carbon dots prepared from ammonium citrate and spermidine have low toxicity and bioaccumulation for zebrafish.

Although the excellent biocompatibility of carbon dots has been recognized, there is still a lack of trial evidence and evaluation criteria for the clinical use of carbon dots. The regulatory status and aspects related to carbon nanomaterials in general and their commercial use are still under discussion and need to be addressed by legislative bodies.

## Carbon dots for diagnosis of viral infections

Infectious diseases have emerged as one of the major cause of morbidity, being responsible for several hundred thousands of deaths per year. Currently, most of the diagnostic testing for viral specimens are still examined in a central laboratories and processed in a batch. Nanoscale material-based analytical tools have a significant effect on turning the current analytical methods into diagnostic approaches by transformation their sensing module for the detection of biomolecules such as viruses. Undoubtedly, contemporary biosensing platforms require simultaneous upgrades because of new challenges (technological limitations and biological barriers) in the diagnosis of viral infections due to fast mutation of viruses. Accurate and rapid detection of viruses is vital for rational and effective therapeutic management at early stages of infection, thus avoiding rapid spread of pathogens from person to person. One of the current strategies is combination of nanomaterials with the traditional viral detection techniques. A common approach to detecting the presence of viruses in biological samples is to use inorganic nanoparticles for single-shot analyses, which are usually solution-based assays performed in a cuvette or a microtiter plate and paper-based lateral flow assays (LFAs). Silver and gold nanoparticles functionalized with protein or nucleic acid probes are ideal for assays with colorimetric detection of viral markers due to their plasmonic properties, while another option is to use them for surface-enhanced Raman spectroscopy (SERS) [63]. In addition, fluorescence-based assays can be designed using, for example, nanoparticles doped with lanthanide chelates or semiconductor QDs [64]. Nanomaterial-based biosensors are devices comprising natural biocompatible materials with a convenient platform for detection of pathogenic organisms. These platforms are usually connected to an appropriate data processing system [65]. Growing interest for virus detection by nanomaterial-based biosensors leads to the bioconjugation of CDs to the biosensor structure. As it was already mentioned, fluorescent CDs offer many fascinating properties, such as chemical inertness, high photostability, easy surface modification, low cytotoxicity, and superior biocompatibility [66–68]. Thus, CDs have attracted considerable attention among scientists as potential candidates in biosensing. Carbon dots possess adjustable elemental compositions and serve as either electron donors or acceptors. These biosensors, which use immobilized biomolecules such as nucleic acids, antibodies, or enzymes for the detection of analytes, convert a biological response into an electrical or optical signal [69]. Suitable integration of CDs as a main elements of virus genome detection methods appears to enhance the sensitivity and specificity for viral recognition. In this section, recent progress in CD-based biosensors for virus detection and diagnosis is reviewed according to which biosensing approach was used.

**Fig. 4:**
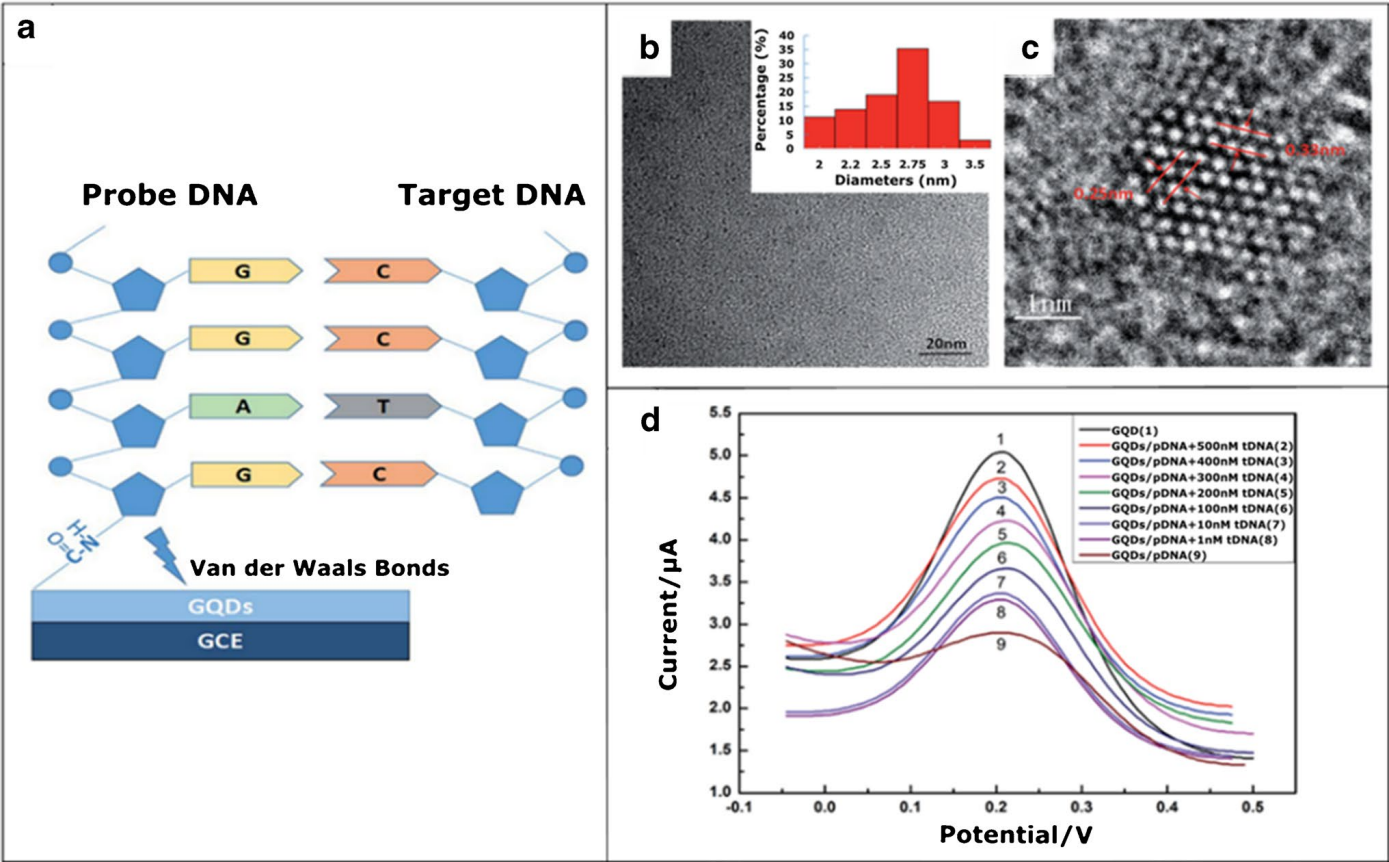
The use of an electrochemical platform for HBV-DNA detection. **a** Electrode arrangement for detection. Probe DNA (pDNA) is loaded onto a GQD-modified GCE and then the target HBV-DNA (tDNA) is immobilized. **b**, **c** TEM and HRTEM images of the GQDs. The inset image is size distribution. **d** Differential pulse voltammogram (DPV) plots. The DPV signals of the GQD-modified GCE after hybridization with different concentrations of HBV-DNA (tDNA). The DPV curves of tDNA detection were obtained by cyclic voltammetry in KCl solution with contained K_3_[Fe(CN)_6_] solution as an electroactive species. *Reprinted with the permission from* [70] *— Published by The Royal Society of Chemistry*

The large surface area to volume ratio and plenty of oxygen-rich functional groups in GQDs, as well as their easy functionalization and excellent electrochemical properties, enable applications of GQDs as surface electrode modifiers and signal amplifiers in the evolution of electrochemical biosensors. The principle of these biosensors is the recognition of target analyte sensed by determining the electric response due to the electrochemical reaction of the target analyte with the surface of the modified electrode of the biosensor. As GQDs are considered as biocompatible, the use of GQD-based nanoprobes is facilitated in biomedical diagnostic areas. A very simple, smart, and efficient electrochemical biosensor based on GQDs, which strongly interact with DNA and play a role of an electrode substance, was introduced by Xiang et al. [70]. In a similar approach, a highly sensitive label-free electrochemical platform using a GQD-modified glassy carbon electrode (GCE; most commonly used as a working electrode in electroanalytical chemistry) coupled with a strongly bound specific sequence of DNA molecules as a probe (pDNA) was designed for the detection of potentially life-threatening hepatitis B virus DNA (HBV-DNA). The GQDs were synthesized by a safe and simple pyrolysis process using citric acid [71]. The mechanism of electron transfer from the pDNA-decorated GQD-modified electrode to the electrochemically active species K_3_[Fe(CN)_6_] was monitored by differential pulse voltammetry (DPV). The low values of peak current caused by the presence of pDNA on the surface of the GQD-modified electrode increased due to the elimination of electrostatic repulsion that occurs after the addition of HBV-DNA, where HBV-DNA-pDNA duplexes were released in a concentration-dependent manner. This strategy demonstrated an excellent linear detection range from 10 to 500 nM and provided a detection limit of only 1 nM, the best value among carbon-based electrochemical DNA biosensors. In addition, it should be noted that this fluorophore-free labeling approach also represents an enzyme-free signal amplification strategy in contrast to standard detection assays. Another advantage is that the biosensor prepared in this way is not only safe and highly sensitive, but also inexpensive. This electrochemical platform for HBV-DNA detection and DPV results are illustrated in Fig. 4.

**Fig. 5:**
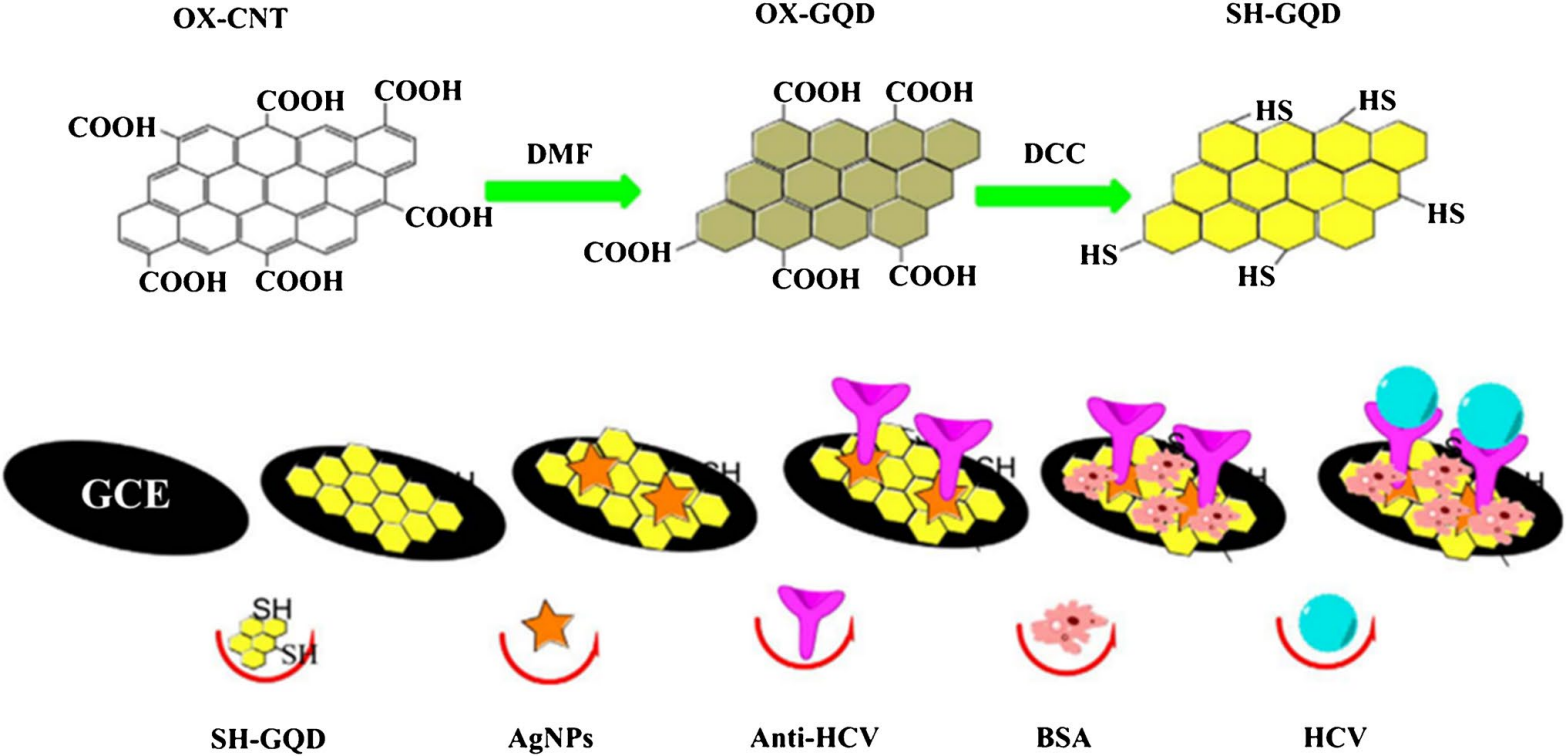
Fabrication process of electrochemical immunosensor constructed for the detection of HCV core antigen involving the use of an AgNPs/SH-GQD nanocomposite as a substratum to immobilize anti-HCV antibody. *Reprinted from* [72]*, Page No. 948, Copyright 2017, with permission from Elsevier*

The use of antigens and their combination with specific antibodies as an efficient strategy has opened new doors for designing of improved assay for hepatitis C infection diagnosis. Compared to conventional immunoassays such as ELISA, which can require several days, electrochemical analyses are much faster. At the same time, key parameters such as signal amplification, sensitivity, and stability must be ensured for accurate sensing. For example, Valipour and Roushani [72] fabricated a label-free electrochemical immunosensor based on a functionalized nanocomposite of silver nanoparticles (AgNPs) decorated with thiolated GQDs (GQD-SH) as GCE modifier for biorecognition of the hepatitis C virus core antigen (HCV). Due to the small size of GQDs, which is less than 100 nm, the resulting signal is amplified and the large surface area allows immobilization of a large number of antibodies on the surface of the nanocomposite. To remove the redox reaction products, the GCE was polished with alumina powder and thoroughly rinsed with deionized water. Subsequently, the electrode was soaked in the GQD-SH solution, followed by the immobilization of AgNPs on the surface of the GQD-SH/GCE via Ag–S bond formation. In the next step, anti-HCV antibody was covalently attached to the surface of AgNPs via its terminal amino group (−NH_2_). In order to experimentally determine the immunorecognition reaction, a biological molecule of riboflavin was used as a redox probe for the detection of HCV using differential pulsed voltammetry measuring. Along with the specific immune recognition, the decrease in the oxidation signal response of riboflavin was investigated. The proposed device showed a wide linear concentration range (0.05 pg mL^−1^ to 60 ng mL^−1^) with the detection limit of 3 fg mL^−1^. Finally, the application of this platform was evaluated also in clinical diagnosis where HCV core antigen was reliably detected in real samples of spiked human serum from patients. This electrochemical device for hepatitis C virus detection is illustrated in Fig. 5. The main feature of GQD-SH in this new type of electrochemical immunosensor is their high surface-to-volume ratio, biocompatibility, dispersibility in relevant solvents, and unique electronic structure that leads to sensitive detection of human serum samples with enhanced signal.

Another sandwich-type electrochemical immunosensor utilizing GQDs was developed for the detection of an oncogenic retrovirus, avian leukosis virus subgroup J (ALVs-J), using apoferritin-encapsulated Cu (Cu-apoferritin) nanoparticles for enhancing of the signal amplitude [73]. GQDs were chemically synthesized via cleaving graphene oxide with the assistance of microwave irradiation [74]. The immunosensor setup involved the Fe_3_O_4_@GQD hybrid platform for the immobilization of secondary ALVs-J antibody (Ab_2_) and Cu-apoferritin nanoparticles as electroactive probe (Fe_3_O_4_@GQD/Ab_2_–Cu-apoferritin). Again, the huge surface area of GQDs prepared by the well-known Hummers’ method enabled immobilization of large number of antibodies on the surface of the immunosensor, resulting in a significant increase in signal. Compared to larger carbon nanotubes, these small particles of 3 to 20 nm exhibit a greater number of carboxyl groups and better conductivity. In addition, their excellent electrocatalytic effect has been observed. In this study, a very nice comparison of the signal amplification abilities of GQDs and apoferritin was investigated. The immunosensor with GQDs showed a larger current shift (Δ*I* 1/4 19.96 μA) than the immunosensor without GQDs. This was mainly attributed to the larger free surface area that GQDs provided for the conjugation of Cu-apoferritin and Ab2. More significantly, the electrochemical immunosensor with apoferritin exhibited a higher peak current (Δ*I* 1/4 47.74 μA) than the immunosensor without apoferritin. These results confirmed that the use of apoferritin can accommodate more electroactive probes and GQDs can immobilize more Cu-apoferritin and Ab2. Therefore, the use of GQDs and apoferritin significantly amplified the immunosensor signal. Deposition of GQDs onto the bare GCE was done using 1-ethyl-3-(3-dimethylaminopropyl)carbodiimide (EDC) and N-hydroxysuccinimide (NHS) as a cross-linker and by subsequent incubation in a primary ALVs-J antibody (Ab_1_) solution. The quantification of the target ALVs-J virus was realized when ALVs-J was captured onto the Ab_1_-immobilized electrode surface, followed by a specific immunorecognition reaction with the Ab_2_ antibody on the hybrid surface with the subsequent formation of the sandwich structure. Electrochemical behavior was evaluated after each step of the surface modification and biosensing studies were conducted by cyclic voltammetry (CV) and DPV, respectively. This electrochemical immunosensor was able to detect as low as 115 TCID_50_ mL^−1^ with a wide detection range from 102.08 to 104.50 TCID_50_ mL^−1^.

New developments in nanotechnology pave the way to robust electrochemical detection of viruses, especially using impedimetric biosensing. Impedimetric-based immunosensors enable to measure the changes in charge conductance and capacitance according to varying concentration in the target analyte immobilized on the immunosensor surface [75]. Applying GQDs as a high-performance material displays promising features for constructing impedimetric biosensors. Moreover, doping of GQDs with chemically bonded nitrogen and sulfur atoms allows to tune their electrochemical properties as well as increasing the amount of the anchoring sites for the adsorption of metal ions. Chowdhury et al. [76] recently reported a discovery of an electrical pulse-induced immunosensor based on the use of nitrogen and sulfur co-doped graphene GQDs (N,S-GQDs) and gold-embedded polyaniline nanowires (AuNP-PAni) for quantifying the hepatitis E virus (HEV) by an impedimetric response. Hepatitis E virus (HEV) infection is known primarily as a cause of acute hepatitis, a viral disease in which even low levels of the virus pose a potentially fatal threat. To fabricate a biosensor electrode, a N,S-GQD@AuNP-PAni nanocomposite—covalently conjugated by the specific anti-HEV antibody—was used, followed by an interfacial self-oxidation-reduction polymerization on a clean GCE. The AuNP-PAni served as enhancers of the electron transfer process and also provided high surface area for the target HEV. The presence of N,S-GQDs on the electrode surface played an important additive role in keeping high conductivity in impedimetric response.

As already mentioned for N,S-GQDs, the chemically bonded nitrogen atom can significantly improve the electrochemical properties by changing the electronic characteristics, and sulfur can increase the number of anchor sites for adsorption on noble metal nanoparticles. N,S-GQDs are also used to enhance the electrochemical activity and conjugation of antibodies through their edge carboxyl groups. By combining the three components, PAni, AuNPs, and N,S-GQDs, a nanocomposite was constructed that has a synergistic effect of its organic and inorganic parts. Therefore, the nanocomposites exhibit excellent electroactivity in analyte solution. To achieve more sensitive detection of HEV, the biosensor was exposed to different external pulses at the time of the virus loading. Such an electrochemical impedance spectroscopy (EIS) biosensing platform could efficiently determine and discriminate various HEV genotypes (G1, G3, G7, and ferret HEV) in wide concentration range from 1 fg mL^−1^ to 100 pg mL^−1^ in cell culture supernatants with a detection limit of 0.8 fg mL^−1^. To confirm the applicability of the method developed in this study, fecal samples of a HEV-infected monkeys were also examined with sensitivity comparable to that achieved by a reverse transcription quantitative real-time polymerase chain reaction (RT-qPCR).

Nowadays, electrochemical biosensors are the most commonly used for detection of various viruses. In spite of the advantages such as low cost, simple instrumentation, and rapid response, this method is sensitive to sample matrix effects, has small temperature range, and short instrument life. Thus, further optimization of this method is proposed.

In the recent past, the range of protein-based virus identification methods was extended by so-called aptamer-based biosensors, also called aptasensors, which consists of a selective and specific aptamer probe displaying robust binding affinity towards the proteinaceous part of viral materials [77]. Aptamers are similar to antibodies, but possess more advantages including greater stability, a smaller size, low cost, and an easier synthesis process. The electrochemical approach which utilizes the aptamer as biorecognition element was introduced, for instance, Ghanbari et al. [78]. They have developed a label-free technique, taking GQD nanocomposite as the electrochemical aptasensor probe for the ultrasensitive quantification of a core antigen from the HCV. To fabricate the aptasensor, GQDs served as an appropriate immobilization substrate for aptamers through π stacking interactions, thus enhancing the aptamer molecule accumulation on the GCE surface. The surface composition of such an aptasensor was monitored by EIS, as well as measuring by CV and DPV techniques after each functionalization step. In this report, the EIS biosensor was able to efficiently determine the target HCV core antigen, which exhibited a wide linear concentration range from 10 to 400 pg mL^−1^ with the detection limit of 3.3 pg mL^−1^. This biosensor probe can efficiently determine the target analyte even in human serum samples.

**Fig. 6:**
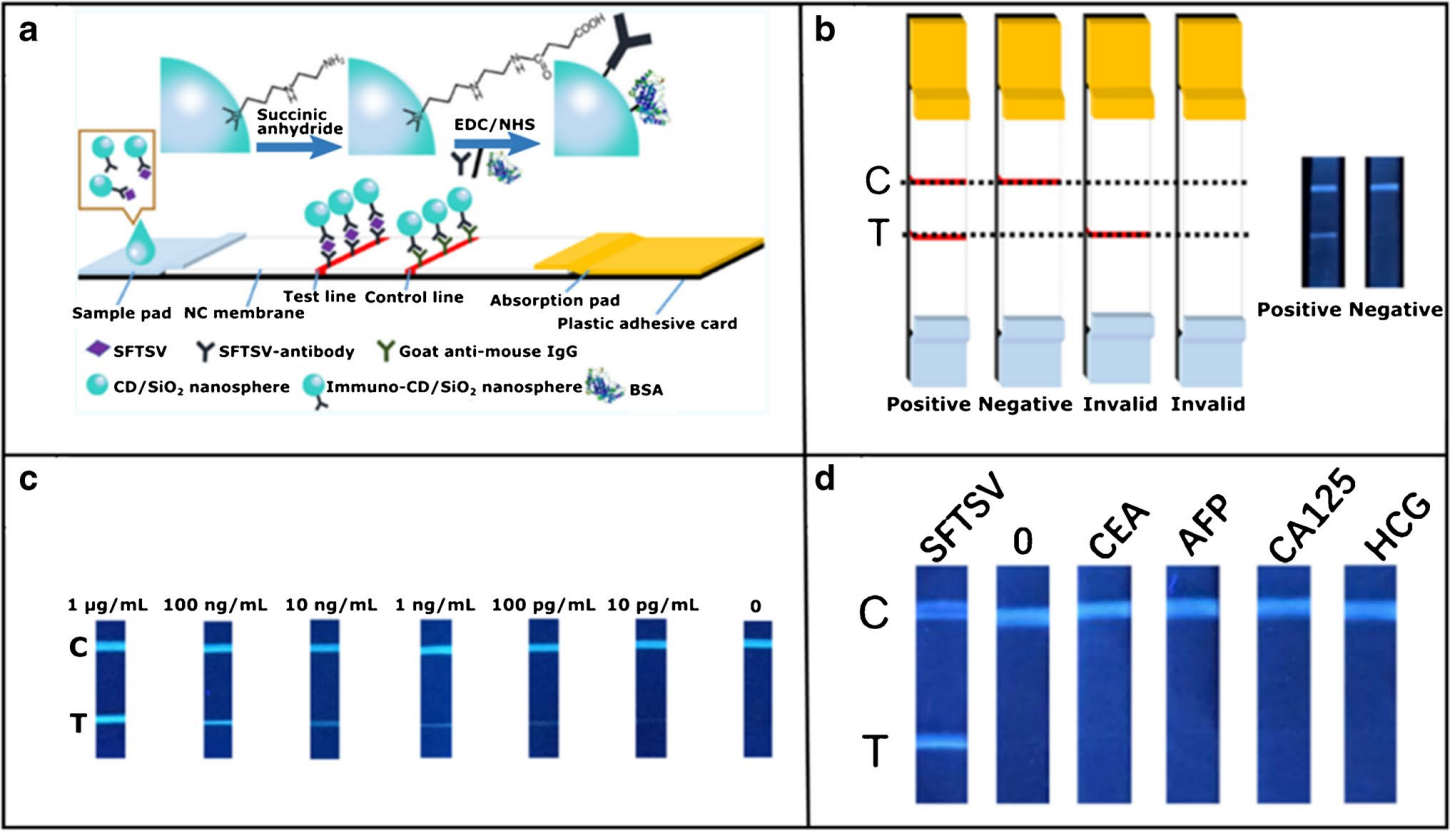
**a** Construction steps of lateral flow test strips for the detection of severe fever with thrombocytopenia syndrome (SFTS) using CDs/SiO_2_ nanospheres (CSNs). **b** Evaluation of results of the immunofluorescent CSNs based test strips. If 2 fluorescence lines show up on the test (T) and control (C) lines, that is a positive result, and negative when only the C line shows up. If only the T line or no line shows up, the test is invalid. **c** Fluorescence pictures of the test strips for investigation of the quantification of SFTSV nanoparticles. The brightness of the T line decreases with decreasing concentration of SFTSV nanoparticles. **d** Evaluation of the selectivity of the method where the blank sample of phosphate buffer and other four different antigens were examined on the strip individually. *Reprinted with permission from* [84]*. Copyright 2019 American Chemical Society*

Among all kinds of biosensor platforms, paper-based biosensors are commercially attractive alternative due to their easy preparation, handling, availability, transportability combined with low-cost and effective manufacture, thus surpassing the screen printed glassy carbon-based electrodes [79, 80]. Lateral flow biosensors do not require sophisticated instrumentation and enable very easy handling. Although a lot of lateral flow test strips have been reported, their sensitivity limits their subsequent application. A sandwich type bioimmunoassay architecture, which is utilized as a unique research tool for the highly biospecific recognition interaction between an antibody and antigen for fast and sensitive key-lock detection, can be also integrated with a lateral flow test strips. Recently, considerable effort has been put in developing a test strip system for the detection of the virus by choosing various labels as a signal reporter such as gold nanoparticles (AuNPs) [81], colored latex beads [82], and quantum dots [83]. Moreover, these test strips can be also coupled with fluorescent CDs as tags to further improve the detection sensitivity. For instance, Xu et al. [84] proposed a point-of-care immunoassay biosensor of fluorescent CD/SiO_2_ nanospheres (CSNs) for the detection of sever fever with thrombocytopenia syndrome virus (SFTSV) with high ultra-sensitivity. The CSNs prepared by a simple co-hydrolysis of silanized CDs with tetraethyl orthosilicate (TEOS) used as a template were coupled with lateral flow test strips. Before the immobilization of the antibody capturing the SFTS virus, the carboxyl-terminated groups of CD/SiO_2_ were activated by using EDC as a coupling agent and NHS as an activator. Due to the excellent fluorescent properties of CSNs, the biosensor provided the benefit of having a longer assay lifetime and good selectivity. Based on this principal, the content of the SFTS virus in buffer was successfully visually observed with a limit of detection 10 pg mL^−1^. Subsequently, four different antigens (HCG, AFP, CEA, and CA125) with a concentration of 1 μg mL^−1^ and the blank sample of phosphate buffer were immobilized individually in five test strips and were used for the examination of the method selectivity. There was no observable any fluorescent intensity of the test line for those nonspecific protein samples, whereas the SFTSV monoclonal antibody specifically recognized the corresponding antigen. Finally, the application of this method was evaluated also in clinical diagnosis where SFTSV was reliably detected in real samples of a patient’s serum. This type of capture assay for SFTS virus is illustrated in Fig. 6.

Later on, in 2021, the same group—Xu et al. [85]—also extended the utilization of the immunofluorescent CD/SiO_2_-based lateral flow strip platforms to sensitive, rapid, and specific detection of Zika virus. The traditional method of Zika virus detection involves amplification of the virus genome, which is time-consuming and requires well-trained technicians. The lateral flow test has been successfully used to detect viruses, cancer, small molecules, bacteria, etc. because of its advantages of immediacy, simplicity, ease of use, and ease of interpretation. Fluorescent CD/SiO_2_ (FCS) structures with various contents of silanized CDs were doped onto the inner surface of tree-like shapes SiO_2_ colloids as the template host via strong Si-O bonding formation. Then, the ZK01 antibody was conjugated with FCS spheres through its carboxyl groups. The immunorecognition reaction was monitored by observing the test results under a 365-nm ultraviolet lamp. The immunosensor showed a wide linear concentration range (10 pg mL^−1^ to 1 μg mL^−1^) with the visual detection limit of 10 pg mL^−1^ and was applied to the analysis of spiked human serum. The measurement of the Zika NS1 protein in a simulated human serum revealed a promising diagnostic applicability. Compared with a traditional AuNP-based lateral flow assay, the results of this FCS-analysis showed 100× higher sensitivity. In addition, Xu et al. used a similar system composed of dendritic silica nanospheres coated with red emissive CDs to detect SARS-CoV-2 nucleocapsid proteins with a sensitivity of 10 pg mL^−1^ and a linear response up to 1 μg mL^−1^ [86]. Moreover, this system can be used in the future as a new and cost-effective simple detection method, especially in poor regions of the world.

**Fig. 7:**
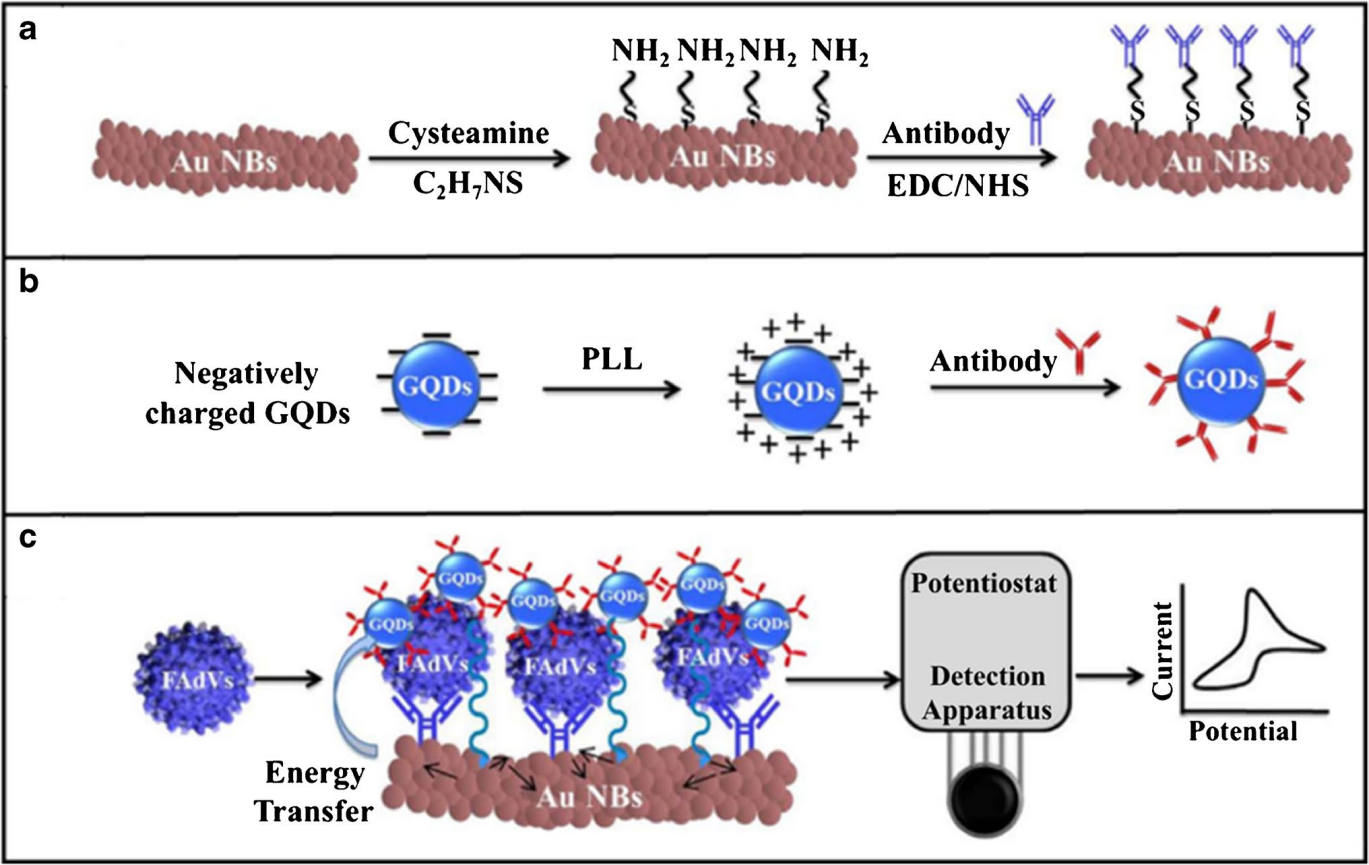
Schematic diagram of virus sensing. a Gold nanobundles (Au NBs) and b GQDs were first conjugated with target-specific antibody, separately. c Then the anti-adenovirus conjugated Au NBs and GQDs formed a nanohybrid structure in the presence of target fowl adenoviruses (FAdVs), resulting in the enhancement of the current peak upon light actively coupling with the matter. *Reproduced with permission from* [92]*, Copyright 2018, Elsevier*

To the best of our knowledge, only one study has recently reported the use of GQD-based photoelectrochemical (PEC) nanobiosensors as a promising low-cost approach for virus detection. When the photoelectrochemical biosensing strategy is adopted, the biological interactions between the analyte and the biorecognition element result in the photoelectric conversion process under light illumination and applied potential where the photocurrent is recorded as the detection signal. Photoelectrochemistry appears to be a challenging research topic because of its remarkably high sensitivity and low background signal, along with being particularly appropriate for biomolecule determination at very low concentrations [87–89]. Moreover, the PEC system possesses specific features such as rapid analytical response, simple instrumentation, and easy miniaturization. The principal of the PEC biosensors consists in the generation of a photocurrent signal during the biological interaction between bioreceptor and the target analyte [88]. This PEC reaction is accompanied with the energy transfer and charge transfer processes under light illumination between the photoactive element and electron donating/accepting moieties [90]. With the progress of nanotechnology, CDs have been used as photoactive species for fabricating PEC biosensors [91]. Again, the large specific surface area and good biocompatibility of CDs provide an opportunity to attach and load large amount of biomolecules and help to maintain their bioactivity significantly. Nevertheless, research in this area is in its infancy, and, thus, the photoelectrochemical mechanism is required to be further examined. In 2018, Ahmed and his team [92] constructed an optoelectronic immunosensor for fowl adenoviruses (FAdVs) identification by exploring the activity of GQDs assembled on a gold nanobundle (Au NB) film. In this work, GQDs were synthesized with the assistance of a benchtop design autoclave. This approach utilized a modified layer-by-layer assembly technology as a promising strategy to the deposition of an Au NB film on a carbon printed electrode through the use of L(+) ascorbic acid, gold chloroauric acid, and poly- _L_ -lysine (PLL). A nanohybrid structure consisting of GQDs and Au NBs was coupled with anti-adenovirus antibody via an electrostatic bonds through positively charged PLL and via an amide bonds through cysteamine, respectively, as depicted in Fig. 7. A local electric signal enhancement under UV light irradiation, where Au NBs and GQDs came close to each other, was directly co-related with the FAdV antigen concentration in the detection ability up to 10 plaque-forming unit (pfu) mL^−1^ and 50 pfu mL^−1^ with the limit of detection (LOD) value of about 8.8 pfu mL^−1^ and 37.2 pfu mL^−1^ in buffer and chicken blood media, respectively. Compared with a traditional gold enzyme-linked immunosorbent assay (ELISA) methods, this optoelectronic biosensing assay provided the excellent sensitivity (100 times higher).

Last but not least is the detection approach using the Golgi apparatus, which was introduced by Li et al. [93]. The method combined the benefits of L-cysteine-functionalized carbon quantum dots (5–10 nm) and silica nanoparticles (40–80 nm), and has been successfully applied for targeting and monitoring the morphological changes of the Golgi apparatus during the process of a viral infection with respiratory syncytial virus under visualization by optical and electron microscope. After the infection, the Golgi broke down into small fragments and become scattered. Here, thanks to the stereo configuration and free thiol groups of _L_-cysteines on their surface, fluorescent carbon quantum dots can target the Golgi with high specificity. Moreover, due to the extraordinary photostability and biocompatibility of these CDs, the system is appropriate for long-term in situ imaging of the Golgi. The authors thus developed a promising approach to the imaging strategy for early diagnosis, drug delivery, and subsequent therapy of Golgi diseases.

Surprisingly, less than 20 papers have been published so far in which CDs have been used to construct biosensors for virus detection (see Table 1). In our opinion, there is still a great opportunity to push forward the effort to develop new breakthrough biosensors based on emerging CDs and their unique optical properties towards fast, sensitive, and specific sensors that could be easily constructed in a low-cost process. Why not have paper strips or antigen assays containing suitably modified CDs where only the very simple “light-on/light-off” detection principle is used to detect, for example, Covid-19?

**Table 1:** Biosensors using CDs for viral detection

Nanomaterials	Technique(s)	Virus detected	Linear range/LOD	Ref.
AgNPs/thiol-CQDs	Electrochemical (DPV)	HCV	0.05 pg mL^−1^–60 ng mL^−1^/3 fg mL^−1^	[72]
N,S-GQDs/AuNPs/PANI	Electrochemical (CV;EIS)	HEV	1 fg mL^−1^–100 pg mL^−1^/0.8 fg mL^−1^	[76]
AuPd/n-GQDs@PS	Amperometry	HBsAg	10 fg mL^−1^–50 ng mL^−1^/3.3 fg mL^−1^	[94]
GQDs/AuNBs Fe3O4/GQDs/Cu-apoferritin GCE/GQDs/DNA GCE/GQDs/aptamer SiO2/CDs/Ab SiO2/CDs/Ab SiO2/CDs/Ab	Electrochemical (CV) Electrochemical (DPV) Electrochemical (CV) Electrochemical (EIS;CV;DPV) Immunofluorescence Immunofluorescence Immunofluorescence	FAdVs ALVs-J HBV HCV SFTSV Zika SARS-CoV-2	10–50 pfu mL^−1^/8.8 pfu mL^−1^ 102.08–104.4 TCID_50_ mL^−1^/115TCID_50_ mL^−1^ 10–500 nM/1 nM 10–70 pg mL^−1^; 70–400 pg mL^−1^/3.3 pg mL^−1^ 10 pg mL^−1^ 10 pg mL^−1^ 10 pg mL^−1^	[92] [73] [70] [78] [84] [85] [86]

## Carbon dots for treatment of viral infections

The use of CDs for the treatment of viral infections is a completely new area of their potential applications. There is only a limited number of publications that elaborate on this problematics. Various approaches were used to face viral infections, whereby CDs and GQDs were utilized either as a delivery platform for other therapeutics or as photodynamically active particles that destroyed the infected cell or as an inhibitor of the binding of the viral particle to the surface of the host cell or virus replication.

**Fig. 8:**
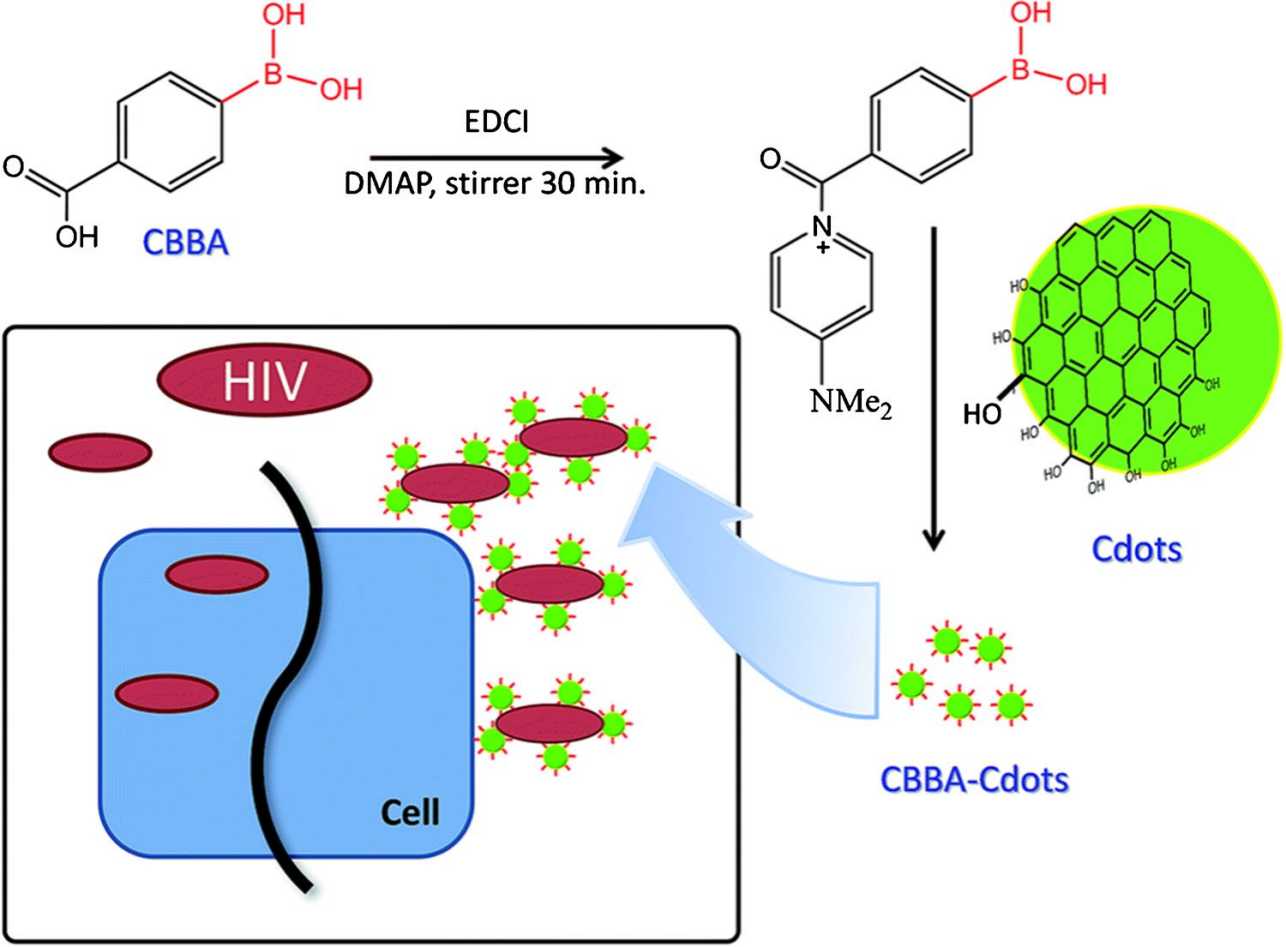
Conjugation of 4-carboxy-3-chlorobenzeneboronic acid (CBBA) with CDs and mechanism of viral entry inhibition by the CBBA-CD conjugates. CBBA-CDs prevent the interaction of the virus with the target cell by forming a tetravalent boronate diester cyclic complex with HIV-1 envelope. *Reproduced with permission from* [96]*, Copyright 2016, Reproduced by permission of The Royal Society of Chemistry*

One of the possible targets is the human immunodeficiency virus HIV-1, which is a retrovirus with its own reverse transcriptase that can be inhibited in order to stop viral replication. It is also spread among the cells through the interaction of viral envelope glycoprotein gp120 with chemokine receptor CD4 and co-receptors CXCR4 in the case of T-trophic HIV-1 strains or CCR5 in the case of M-trophic strains, or it can use both co-receptors when the dual-trophic strain has evolved [95]. Blocking this interaction can prevent infection of the target cell. Two recent studies presented the anti-HIV activity of CDs; each study used different types of CDs and a different mechanism of action. In the first study, CDs were synthesized by calcination of anhydrous citric acid at 250 °C for 30 min. After neutralization with NaOH, the CDs were purified by dialysis, and the material was further modified with 4-carboxy-3-chlorobenzeneboronic acid (CBBA) using 1-ethyl-3-(3-dimethylaminopropyl) carbodiimide (EDC). The resulting negatively charged CBBA-CDs had a hydrodynamic diameter of 6.2 nm and were proven nontoxic to MOLT-4 human leukemia cells up to 300 μg mL^−1^. The in vitro anti-HIV activity of CBBA-CDs was monitored by syncitia formation in MOLT-4 and MT4/HIV-1 (MT4 cells persistently infected with HIV-1) co-culture where syncitia formation represented the cell-to-cell CD4/CXCR4-dependent pathway of HIV-1 spread. The authors showed that the syncitia formation was effectively prevented by incubating the co-culture with 300 μg mL^−1^ of CBBA-CDs, which did not happen with CDs alone. The proposed mechanism of action is that boronic acid residues bind to the envelope of HIV-1 by the formation of tetravalent boronate diester cyclic complex with 1,2-*cis* diol sites of gp120, thus preventing the interaction of HIV-1 with the target cell receptor CD4 and co-receptor CXCR4 (Fig. 8). The authors also demonstrated that bare CDs had a weak inhibitory effect on the MT4/HIV-1 cell after 24-h incubation (IC_50_ = 9506.3 μg mL^−1^), which was probably due to non-covalent interactions of hydroxyl and carboxylate residues of the CDs with the HIV-1 envelope; however, the inhibitory effect of CBBA-CDs was significantly higher (IC_50_ = 26.7 μg mL^−1^). The study did not address the virus-to-cell pathway; however, both pathways are known to be dependent on the binding of gp120 to the surface receptor of the host cell and the cell-to-cell transmission proved to be a dominant way of the infection propagation [96].

**Fig. 9:**
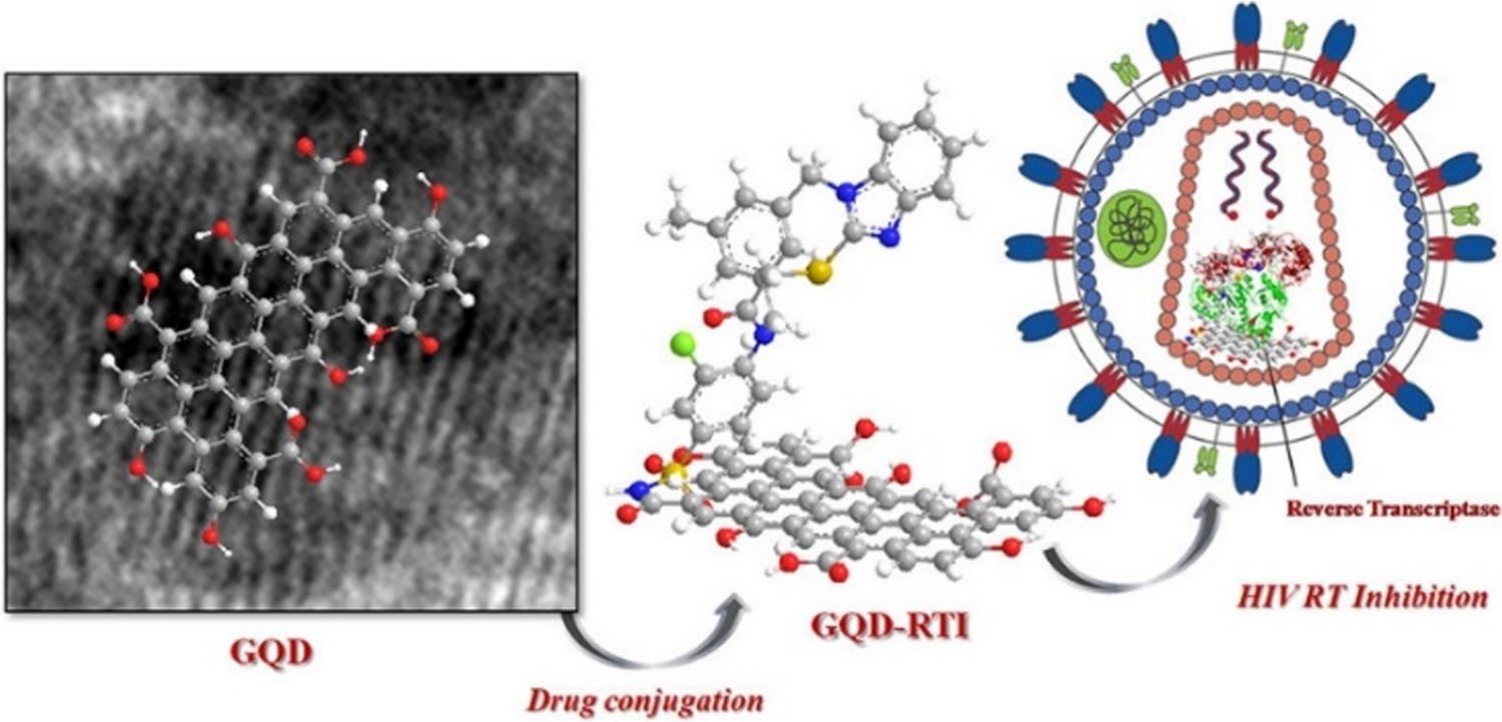
Conjugation of GQDs with reverse transcriptase (RT) inhibitor CHI499 and targeting the HIV-1 RT. Upon treatment, the unstable imide bond between GQD and CHI499 is cleaved and GQDs interferes with the cell-virus interaction, while free CHI499 inhibits viral RT. *Reprinted with permission from* [97]*, Page No.3084, Copyright 2018 American Chemical Society*

In the second study, the HIV-1 virus was targeted with GQDs prepared by acidic oxidation and exfoliation of multiwalled carbon nanotubes and their conjugates with two types of non-nucleoside reverse transcriptase inhibitors (NNRTI), CDF119 or CHI499 (Fig. 9). The authors performed in vitro RT inhibition assay with the GQDs, the drugs, and the conjugates. They showed that the GQDs inhibited the RT reaction with IC_50_ = 37.6 μg mL^−1^, while the more potent drug—CHI499—with IC_50_ = 0.67 μg mL^−1^. The IC_50_ of the most promising conjugate GQD-CHI499 was measured to be 0.09 μg mL^−1^. Other measurements were performed to study the effective concentrations (EC) of the presented systems that inhibited the HIV-1-mediated cytopathic effect, as well as the cytotoxic concentrations (CC) in the MT4 cell cultures. They demonstrated that GQDs alone had EC_50_ of >19.9 μg mL^−1^ with the EC_50_/CC_50_ ratio lower than three. However, the GQD-CHI499 conjugate showed EC_50_ of 0.066 μg mL^−1^ and the EC_50_/CC_50_ ratio of 362, while the values for CHI499 alone were EC_50_ = 0.12 μg mL^−1^ and EC_50_/CC_50_ = 540. The authors propose that the GQD-CHI499 conjugate has a dual mechanism of action, where the drug CHI499, which is bound by cleavable imide bond, is released from the GQDs and inhibits the RT, while GQDs block the virus-cell binding. This explains why the GQD-CHI499 has lower EC_50_ than the drug alone. By contrast, the GQD-CDF119 conjugate has much worse properties than free CDF119 because there is no release of the drug from the system due to stable amide bonding between the GQDs and CDF119. Apparently, the GQD-CHI499 conjugate is the most promising candidate for HIV treatment in this study [97].

**Fig. 10:**
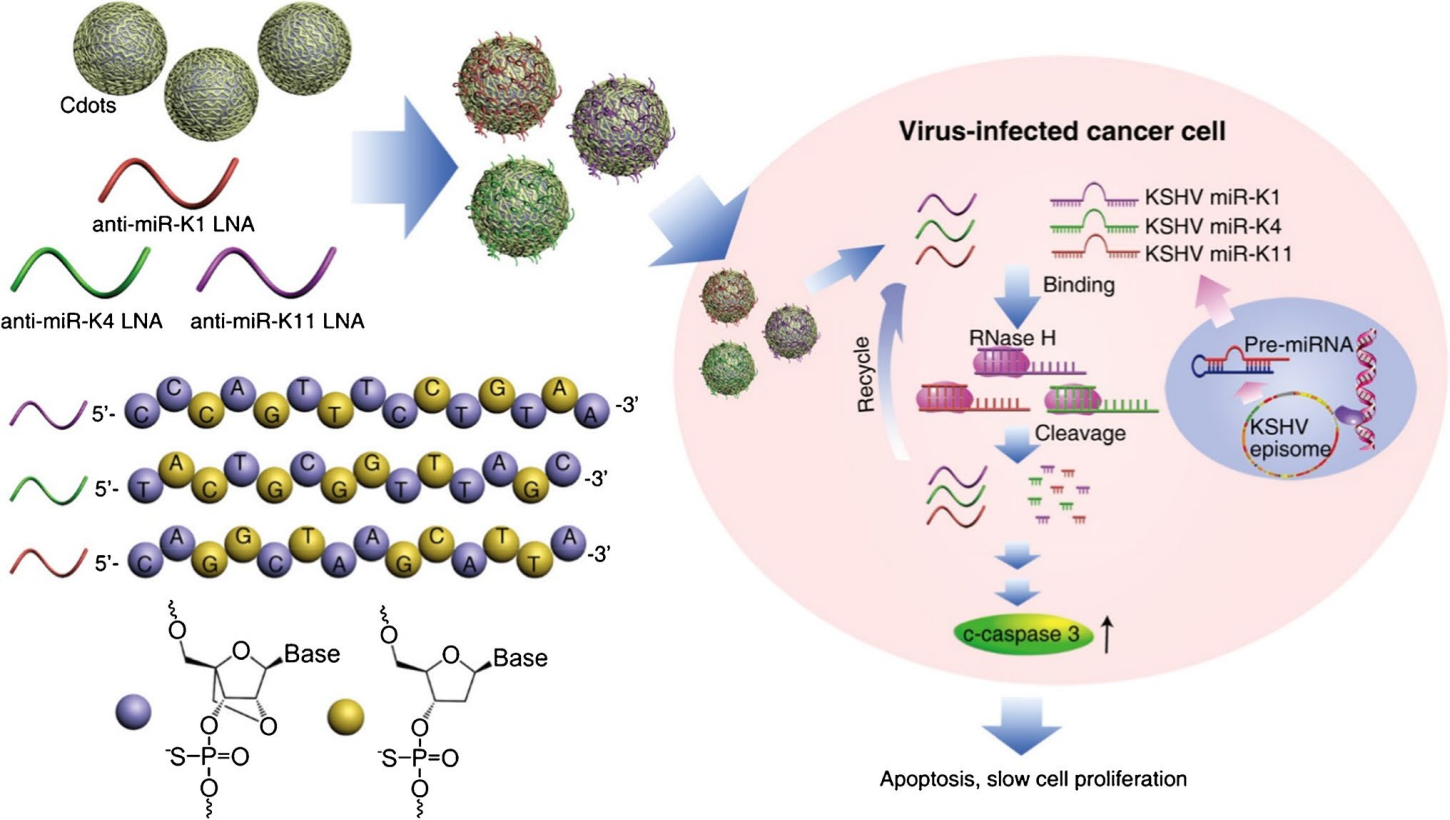
Scheme of the targeting KSHV-encoded miRNAs by LNA-loaded CDs. Locked nucleic acid oligonucleotides are delivered to the virus-infected cancer cell via carbon dots, where these LNA oligonucleotides specifically knockdown viral miRNAs, resulting in the cessation of replication of the virus-infected cancer cell. *Reprinted with permission from* [98]*, Page No. 477. Copyright 2020 American Chemical Society*

Carbon dots can deliver not only “classical” drugs (e.g., enzyme inhibitors) but also therapeutic nucleic acids. In a recent study by Ju et al. [98], CDs prepared by microwave-assisted pyrolysis of polyethyleneimine (PEI) and citric acid were used to deliver a therapeutic locked nucleic acid (LNA) oligonucleotides to cancer cells induced by oncogenic Kaposi’s sarcoma-associated herpesvirus (KSHV), so-called KSHV-associated primary effusion lymphoma (PEL) cells. This virus is known for encoding microRNAs (miRNAs) that cause uncontrolled cell proliferation and such miRNAs are a potential target for an effective treatment. The CDs-LNAs complexes were designed to specifically knockdown KSHV miRNA miR-K12-1, miR-K12-4, and miR-K12-11, which consequently induces apoptosis and stops the proliferation of PEL cells through the activation of cleaved caspase 3 (Fig. 10). Additionally, the authors demonstrated that CDs-LNAs were capable of blocking the initiation of primary effusion lymphoma in xenograft mice, and, moreover, it also triggered tumor regression in mice with fully established PEL. The treatment led to a marked increase in the survival rate of the mice.

Antiviral activity against a pseudorabies virus (PRV) and a porcine reproductive and respiratory syndrome virus (PRRSV) was observed with CDs derived from PEG-diamine and ascorbic acid mixture prepared by the solid-phase thermal reaction. These CDs did not show any toxic effects on MARC-145 cells up to 250 μg mL^−1^ after 48-h incubation and only a slight decrease in viability of PK-15 cells was observed under the same conditions. Models PRV and PRRSV represent the families of DNA and RNA viruses, respectively. First, the authors performed a plaque assay to monitor both intracellular and extracellular virus titers. They infected SK-15 cells with PRV and MARC-145 cells with PRRSV and, in both cases, a significant decrease in the virus titers was observed after 12 h, 18 h, and 24 h post-infection in cells that were incubated with 125 μg mL^−1^ of CDs, when compared to the untreated cells. To support these results, the expression of viral protein markers PRV gD, PRV VP16, and PRRSV-N was monitored by indirect immunofluorescence and western blotting. Again, a significant decrease in the expression of these markers was observed with CD-treated cells. To elucidate the mechanism of action, the innate immune response was tested using MARC-145 cells that were stimulated with CDs at a concentration of 125 μg mL^−1^. It was shown by mRNA quantification that the incubation with CDs caused the stimulation of interferon-α (IFN-α) expression, which led to a cascade where expression of hundreds of IFN-stimulated genes (ISGs) was induced. This phenomenon was demonstrated by the increase in the mRNAs of three ISGs, namely ISG-15, ISG-54, and IP-10 upon the exposure of the MARC-145 cells to CDs. The activation of the type I interferon response then led to the inhibition of viral replication [99]. Additionally, the authors prepared, by hydrothermal synthesis, another two types of biocompatible CDs that displayed blue (b-CDs) and cyan (c-CDs) fluorescence. These CDs inhibited PRV in a way similar to that of the CDs mentioned above. Moreover, the b-CDs were able to selectively enter the cell cytoplasm, while the c-CDs entered the whole cell including the nucleus, which opens the door to selective cell imaging [100].

Additionally to HIV-1, boronic acid-derived CDs could be used to combat herpes simplex virus type 1 (HSV-1), as shown by Barras et al. Here, three types of functional CDs were prepared by hydrothermal carbonization using different precursors, namely phenylboronic acid (B-CDs), 3-aminophenylboronic acid (3-AB-CDs), and 4-aminophenylboronic acid (4-AB-CDs). After the synthesis, large precipitates were removed by centrifugation and the supernatant was purified by dialysis against water. To measure the in vitro toxicity of the final products, Vero cells (monkey kidney cancer) and A549 cells (human lung cancer) were incubated for 24 h and then the CD samples were added for 2 h, followed by washing with PBS and incubating for another 72 h. Finally, the viability was measured using a resazurin assay. It was observed that all three types of CDs were nontoxic to the A549 cells up to the concentration of 300 μg mL^−1^. The Vero cells also did not show any significant drop in viability after the incubation with B-CDs and 4-AB-CDs; however, 3-AB-CDs showed some toxicity at the highest concentration. In the antiviral assay, B-CDs did not show any anti-HSV-1 activity as both infected cell lines lost most of their viability after the procedure. The other two types of CDs showed promising results as there was a concentration-dependent anti-HSV-1 effect. In the case of 3-AB-CDs, the EC_50_ was 0.424 μg mL^−1^ for the A549 cells and 0.469 μg mL^−1^ for the Vero cells. 4-AB-CDs displayed even better properties with EC_50_ of 0.145 μg mL^−1^ for the A549 cells and 0.080 μg mL^−1^ for the Vero cells. Based on a Vero/HSV-1 in vitro model, the mechanism of action of CDs has yet to be fully understood. Nevertheless, it was shown that boronic acid residues are not involved in the virus entry inhibition. More likely, the CDs block the virus-cell interaction in the early stage of the infection by interacting with the cell surface via its NH_2_ and COOH functionalities, while this interaction is also dependent on the size of the CDs. This hypothesis is further supported by the findings that, unlike the effective 3-AB-CDs and 4-AB-CDs, the ineffective, 3–6 times bigger, B-CDs do not interact with the surface of Vero cells [101].

Dong and co-authors [102] reported potential antiviral activity of CDs with two different coatings, uncharged CDs passivated with 3-ethoxypropylamine (EPA) and positively charged CDs passivated with 2,2′-(ethylenedioxy)bis(ethylamine) (EDA). As a model system, they used Norovirus (NoV) virus-like particles (VLPs) of two strains, GI.1 and GII.4. The authors demonstrated the ability of both types of CDs to inhibit the interaction of NoV VLPs to saliva histo-blood group antigen (HBGA) receptors and corresponding anti-VLP antibodies. The blocking of VLPs-HBGA interaction was much more effective than that of the VLPs-Ab binding and the positively charged EDA-CDs were shown to block both interactions stronger than the uncharged EPA-CDs. This was most likely due to the negative charge of the VLP surface. Furthermore, the capsid proteins of VLPs were shown to stay intact after the treatments with CDs, which was demonstrated by western blotting and transmission electron microscopy.

**Fig. 11:**
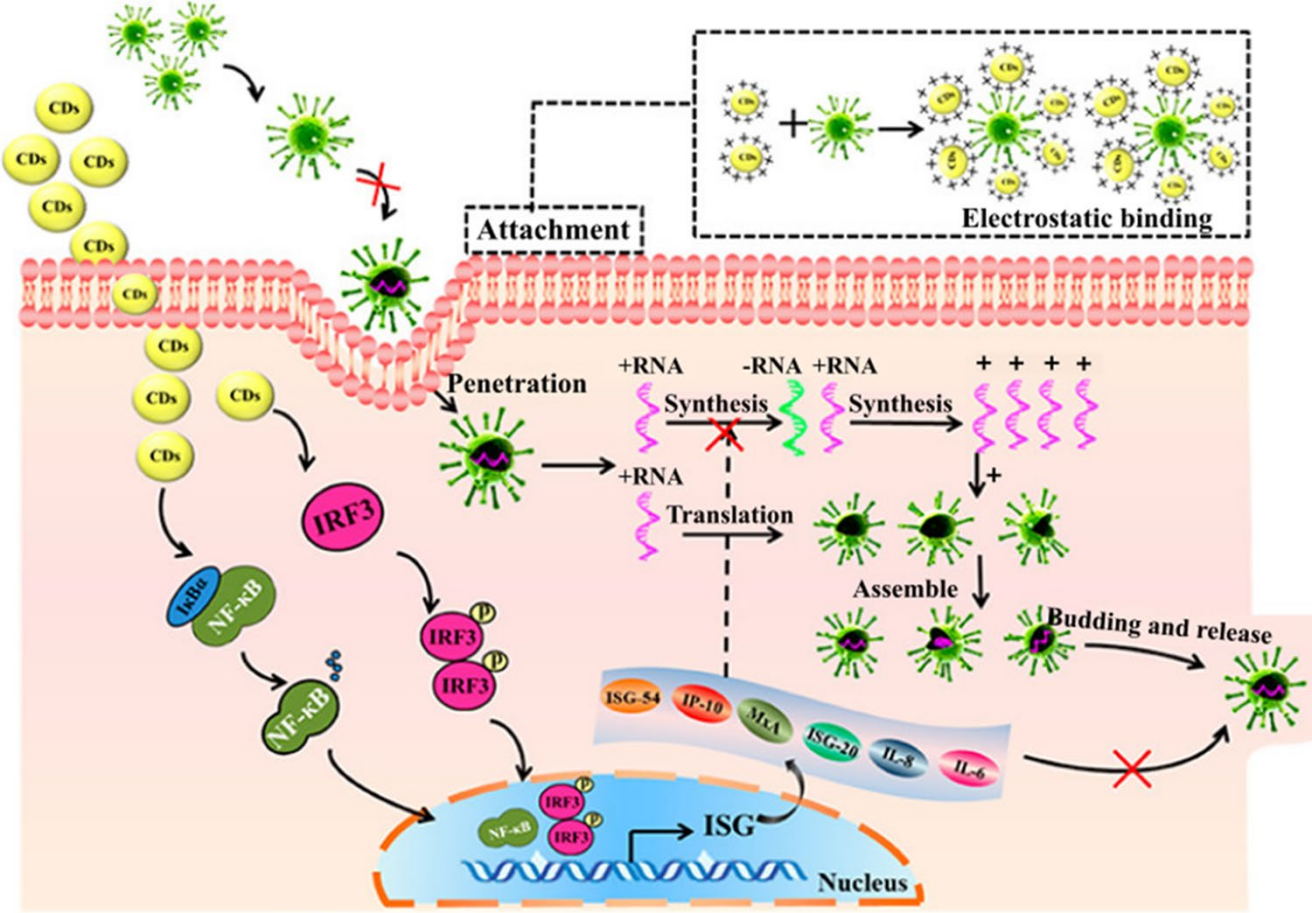
Viral inhibition mechanism of curcumin-derived (CCM) CDs. CCM-CD counteracts the PED virus by several mechanisms. The first is their electrostatic interaction with the viral particle, which leads to inhibition of virus entry and also inhibition of negative-strand viral RNA synthesis. Other mechanisms include suppression of ROS induced by PEDV and stimulation of ISG and pro-inflammatory cytokines. *Reprinted with permission from* [103]*, Page No. 5451. Copyright 2018 American Chemical Society*

**Fig. 12:**
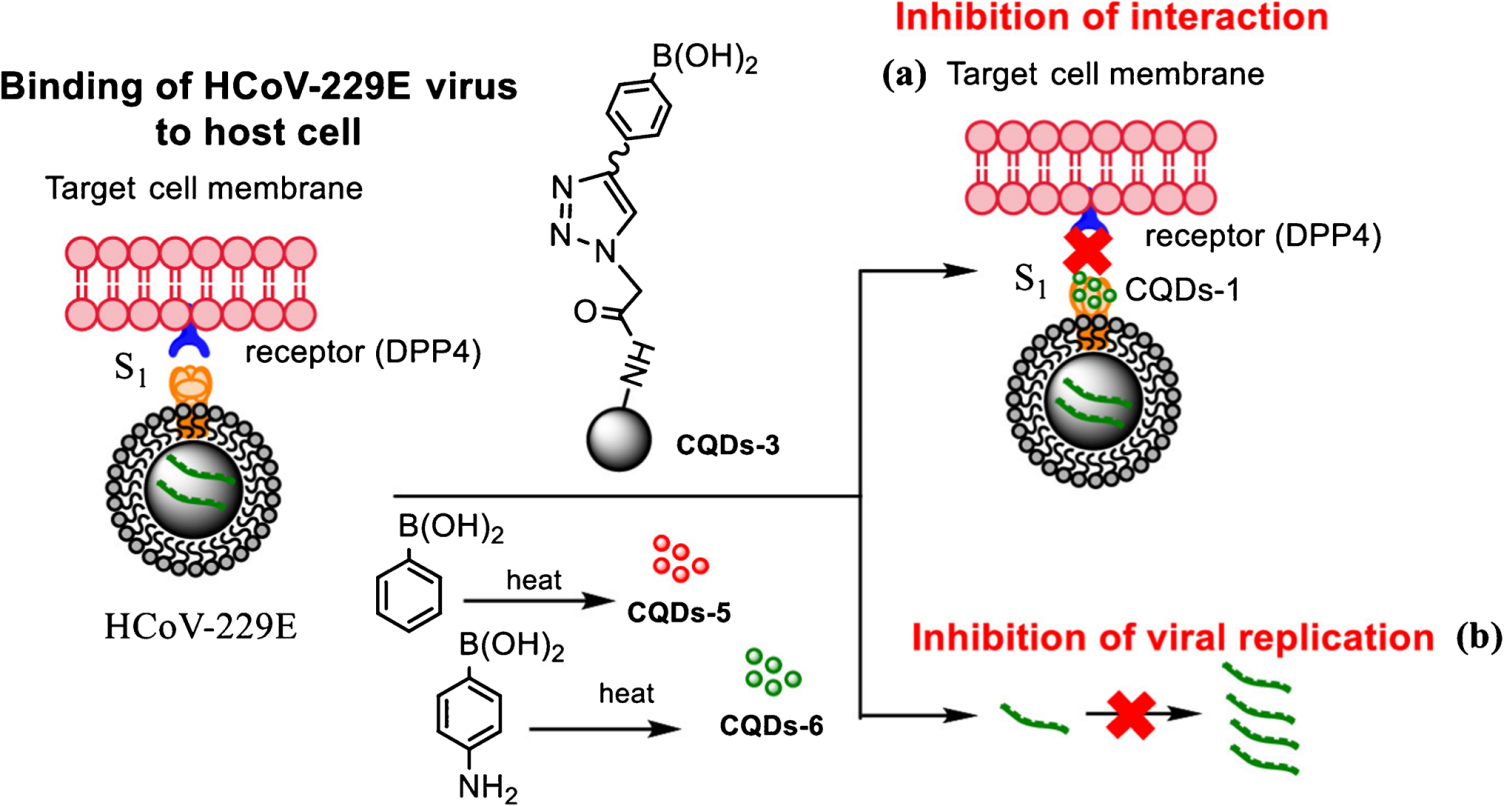
Synthesis of CDs and their mechanisms of inhibition of HCoV-229E virus. These CDs block the virus-cell interaction (a) and inhibit viral genomic RNA replication (b). *Reprinted with permission from* [104]*, Page No. 42965. Copyright 2019 American Chemical Society*

Since its onset in 2019, the COVID-19 pandemic has not been combatted yet; therefore, it is highly desirable to search for drugs that will target coronaviruses. One of the potential candidates is cationic CDs prepared by pyrolysis of curcumin (CCM-CDs) which were used to suppress porcine epidemic diarrhea virus (PEDV). This material can alter the structure of a viral surface protein by electrostatic interaction with the virus, thus inhibiting the viral entry and preventing the synthesis of the viral negative-strand RNA. CCM-CDs also act as a suppressor of reactive oxygen species (ROS) that originate in the PEDV presence. Their mechanism of action also includes stimulation of ISGs and proinflammatory cytokines in Vero cells (Fig. 11) [103]. Human coronavirus HCoV-229E was also subjected to treatment by seven different types of CDs that were divided into two groups. Carbon dots of the first group were prepared by hydrothermal carbonization of ethylenediamine/citric acid mixture and further modified with boronic acid ligands. The second-generation CDs were prepared directly by carbonization of 4-aminophenyl boronic acid with no further modifications. The EC_50_ values were 52 μg mL^−1^ for the first generation and 5.2 μg mL^−1^ for the second generation, while the dual mechanism of action involved the virus replication blockade and the inhibition of the viral entry through the interaction of CDs with the cellular receptors (Fig. 12) [104]. Additionally, one-step hydrothermal synthesis of nitrogen and boron co-doped CDs from citric acid, a quite commonly used precursor for CD preparation, mixed with p-phenylenediamine and sodium tetraborate, can potentially act as effective inhibitors of human coronaviruses [105].

**Fig. 13:**
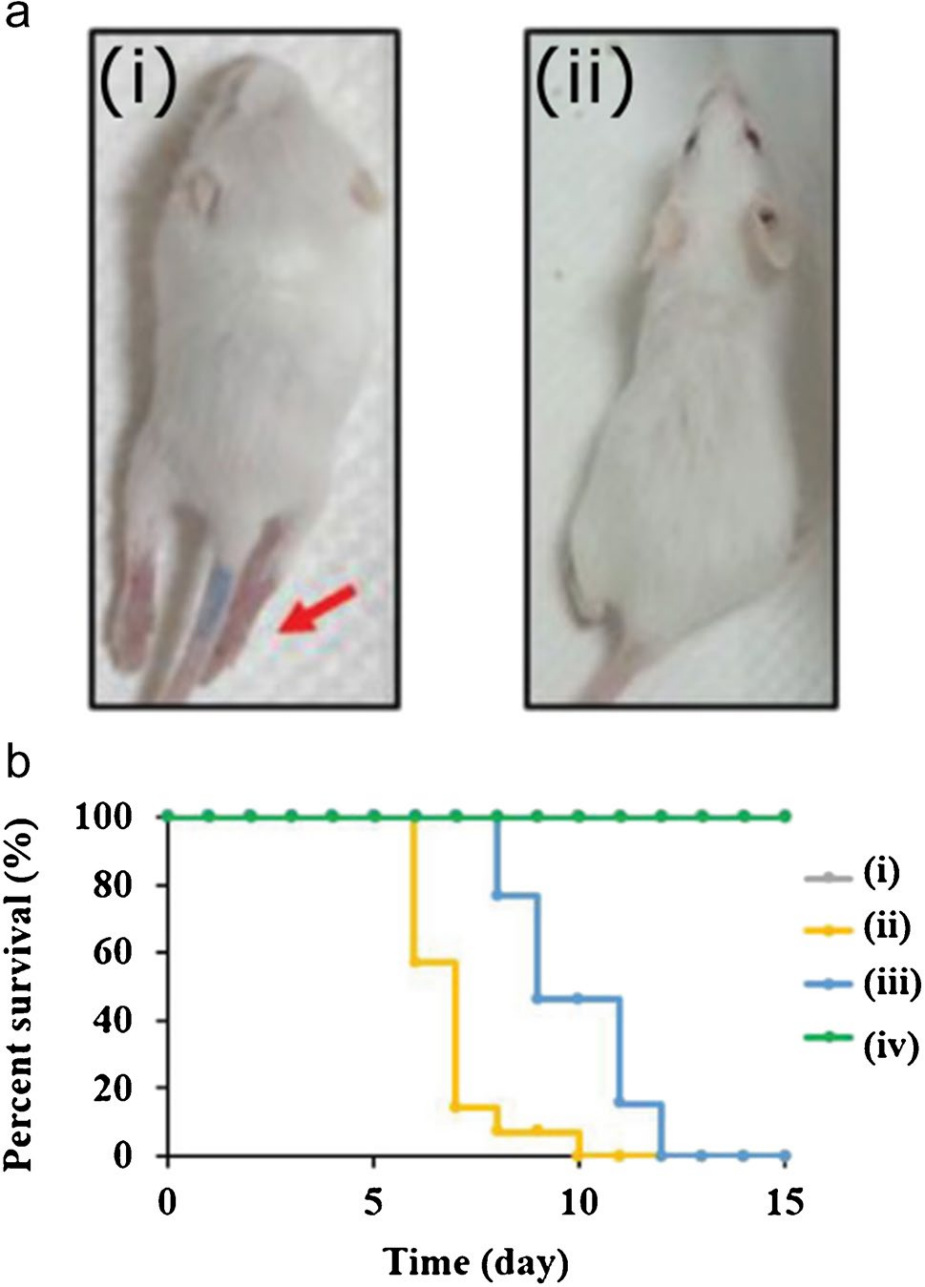
a Photograph of mice 7 days post-infection with enterovirus 71 (i) without and (ii) with treatment with Cur-CDs (25 mg kg^−1^). The red arrow indicates limb paralysis in the untreated mice. b Survival rates of mice (i) without infection and infected mice intraperitoneally injected with (ii) PBS, (iii) curcumin (25 mg kg^−1^), and (iv) Cur-CDs (25 mg kg^−1^). *Reproduced with permission from* [106]*, Copyright 2019, Wiley-VCH*

As we have previously seen [103], curcumin-derived CDs possess interesting antiviral properties. Lin et al. [106] developed another type of Cur-CDs showing activity against enterovirus 71 (EV71) with EC_50_ as low as 0.2 μg mL^−1^ while preserving high biocompatibility to RD cells (CC_50_ = 452.2 μg mL^−1^). Moreover, it was demonstrated by in vivo studies that the administration of Cur-CDs into newborn mice significantly decreased their mortality and prevented hind-limb paralysis caused by EV71 (Fig. 13).

Since multiple viruses cause millions of infections and deaths every year, there is an urgent call for the discovery of broad-spectrum antiviral drugs. As published by Huang et al. [107], benzoxazine monomer-derived CDs could offer a highly desirable solution. The so-called BZM-CDs were prepared by the hydrothermal procedure under alkaline conditions and exhibited activity against various types of flaviviruses (e.g., Zika, dengue, and Japanese encephalitis viruses) as well as non-enveloped viruses (e.g., porcine parvovirus and adenovirus-associated virus) in cell culture experiments. Moreover, an excellent in vitro biocompatibility to Vero and BHK-21 cells was observed after a treatment with BZM-CDs at a concentration of 75 μg mL^−1^. The mechanism behind the antiviral properties of BZM-CDs is that the material interacts directly with virions, thus limiting the virus transmission. The advantage of such a type of mechanism consists in a potentially broad spectrum of targeted viruses, including the newly revealed ones.

Another example of a potentially wide-spectrum antiviral drug is highly biocompatible Gly-CDs prepared from glycyrrhizic acid by hydrothermal synthesis under alkaline conditions. It is already known that glycyrrhizic acid itself possesses antiviral properties via its sugar moiety [108]. These glycyrrhizic acid-derived CDs display a multimodal mechanism of action as it triggers the innate immune response by the stimulation of IFN and ISGs and, similarly to curcumin-derived CDs, Gly-CDs also hinder the accumulation of cellular ROS. Gly-CDs proved an excellent activity against PRRSV, but were also found active against PRV and PEDV viruses [109].

**Fig. 14:**
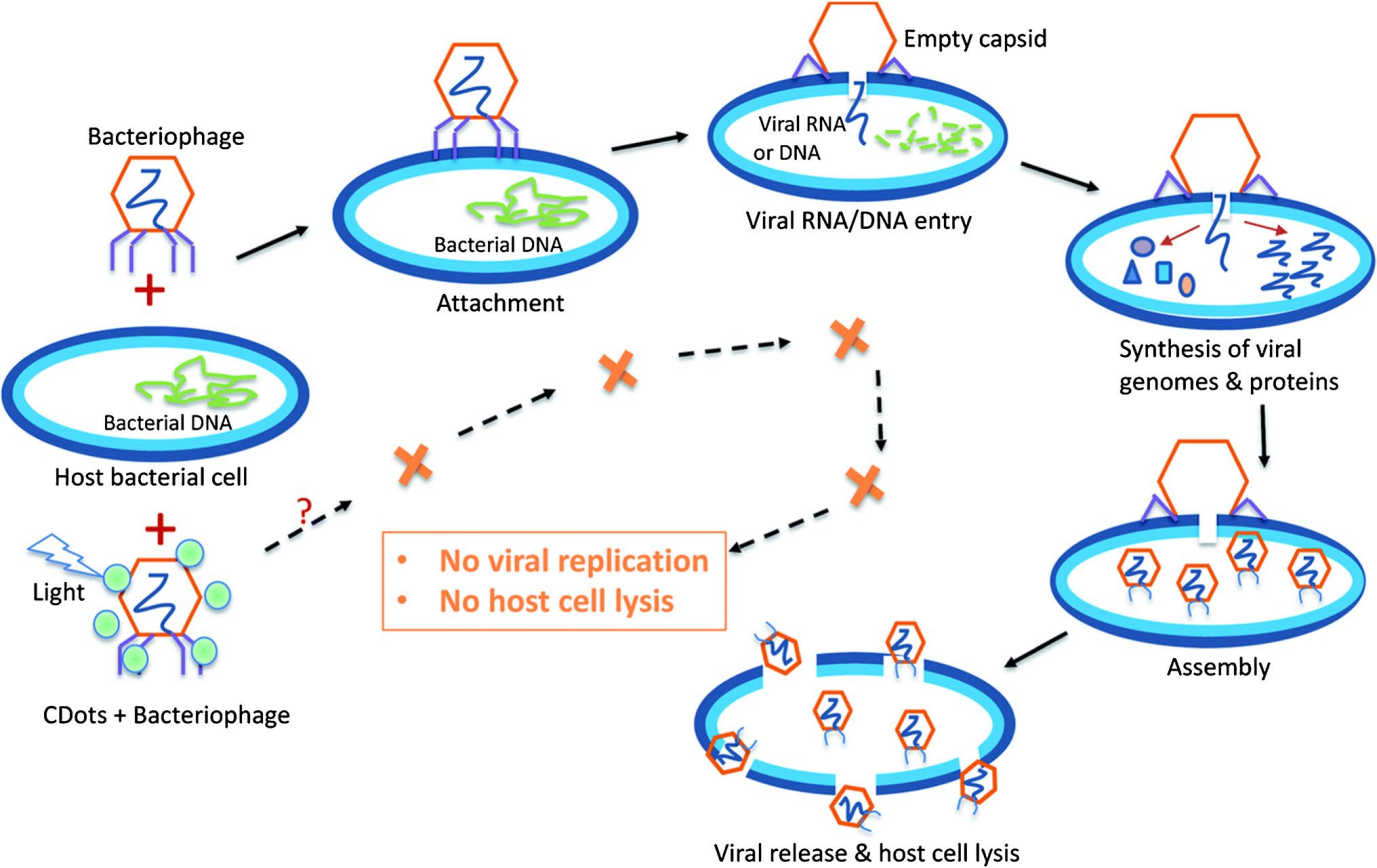
Life cycle of a bacteriophage and expected effect of photodynamic virus inactivation by CDs, which lead to the carbonylation of surface protein and degradation of genomic RNA of the bacteriophage MS2. *Reproduced with permission from* [110]*, Page No. 33945, Copyright 2020. Reproduced by permission of The Royal Society of Chemistry*

Carbon dots with a visible light-activated antiviral activity were introduced by Dong et al. [110]. These CDs were prepared from commercial carbon nano-powder and further modified with EDA. When photoexcited, they exhibited activity against bacteriophage MS2 while no significant altering of the integrity and morphology of the bacteriophage was observed. The photodynamic inactivation of the MS2 consisted of surface protein carbonylation and degradation of genomic RNA (Fig. 14). Since the antiviral effect was quenched by the addition of _L_-histidine (singlet oxygen scavenger), and not by *tert-*butanol (hydroxyl radical scavenger), the singlet oxygen probably played a role in the MS2 bacteriophage photodynamic inactivation. However, the role of different mechanisms of _L_-histidine-dependent antiviral activity quenching, as well as the role of short-lived hydroxyl radicals, cannot be ruled out, and further investigation will be needed to fully understand the exact mechanism of action.

Emam et al. [111] investigated the antiviral effects against coronavirus MERS-CoV of two types of CDs prepared by a facile, green, and low-cost hydrothermal procedure. They compared the biocompatibility and antiviral performance of pullulan-based CDs and CDs based on carrageenan, and found out that carrageenan-derived CDs were preferable due to lower cytotoxicity while retaining their antiviral activity, which was comparable to that of pullulan-derived CDs. Unfortunately, the authors did not investigate the mechanism of antiviral action of both types of CDs.

It is noteworthy that CDs could find applications also in aquaculture and agriculture. Polyamine CDs could potentially be useful as an additive to feed cultured shrimps in order to prevent the white spot syndrome (WSS) virus infection, which leads to severe mortality rates of shrimps. The study revealed that CDs managed to boost innate immunity by affecting the expression of various genes and inhibited the WSSV infection, likely due to its attachment to the viral envelope [112]. Another type of biocompatible CDs showed promising properties as an adjuvant in g85 protein-based vaccine against avian leukosis virus subgroup J (ALV-J) for use in chickens. The CDs-supplemented vaccine led to higher levels of desirable antibodies compared to the same vaccine combined with commercial Freund’s adjuvant. The authors attributed these results to the high protein-leading efficiency of CDs and improved stability of the vaccine [113]. The overview of antiviral carbon is summarized in Table 2.

**Table 2:** Summary of antiviral carbon dots

Virus	Species	Carbon dots type	Synthesis/precursor	Mechanism of action	Reference
HIV-1	*Human immunodeficiency virus 1*	Boronic acid-functionalized CDs	Pyrolysis/citric acid	Virus entry inhibition	[96]
HIV-1	*Human immunodeficiency virus 1*	RTI-conjugated CDs	Acidic oxidation and exfoliation/MWCNTs	RT inhibition, virus entry inhibition	[97]
KSHV	*Human gammaherpesvirus 8*	LNAs-conjugated CDs	Pyrolysis/PEI, citric acid	Viral miRNAs knock-down, virus-induced cancer cell eradication	[98]
PRV PRRSV	*Suid alphaherpesvirus 1* *Betaarterivirus suid 1*	PEG-diamine/citric acid CDs	Solid-phase thermal reaction/PEG-diamine, citric acid	Stimulation of IFN-α and ISG expression	[99]
PRV	*Suid alphaherpesvirus 1*	Blue- and cyan-fluorescent CDs	Hydrothermal/young barley leaves, citric acid, urea	Stimulation of IFN-α, IFN-β, and ISG expression	[100]
HSV-1	*Human alphaherpesvirus 1*	Boronic acid- and amine-functionalized CDs	Hydrothermal/3- or 4-aminophenylboronic acid	Virus entry inhibition	[101]
NoV	NoV VLPs	EPA- and EDA-passivated CDs	CD passivation by 2,2′-(ethylenedioxy)bis(ethylamine) or 3-ethoxypropylamine	VLP binding inhibition	[102]
PEDV	*Porcine epidemic diarrhea virus*	Cationic curcumin-derived CDs	Pyrolysis/curcumin	Inhibition of virus entry and replication, ROS suppression, ISG stimulation	[103]
HCoV-229E	*Human coronavirus 229E*	Boronic acid CDs	Hydrothermal/ethylenediamine, citric acid or 4-aminophenylboronic acid	Inhibition of virus entry and replication	[104]
EV71	*Enterovirus A71*	Curcumin CDs	Solid state pyrolysis/curcumin	Inhibition of virus entry and replication	[106]
JEV ZIKV DENV AAV PPD	*Japanese Encephalitis virus* *Zika virus* *Dengue virus* *Adeno-associated dependoparvovirus* *Ungulate protoparvovirus 1*	Benzoxazine CDs	Hydrothermal/benzoxazine monomer	Virus entry inhibition	[107]
PRRSV PRV PEDV	*Betaarterivirus suid 1* *Suid alphaherpesvirus 1* *Porcine epidemic diarrhea virus*	Glycyrrhizic acid CDs	Hydrothermal/glycyrrhizic acid	Stimulation of IFN and ISG expression, ROS suppression	[109]
MS2	*Emesvirus zinderi*	EDA-functionalized CDs	CDs functionalization with 2,2′-(ethylenedioxy)bis(ethylamine)	Photodynamic inactivation of virus	[110]
MERS-CoV	*Middle East respiratory syndrome-related coronavirus*	Carrageenan- and pullulan-derived CDs	Hydrothermal/carrageenan, pullulan	-	[111]
WSSV	*White spot syndrome virus*	Polyamine CDs	Pyrolysis/spermidine	-	[112, 114]

## Conclusion and perspectives

Carbon dots are a kind of nanomaterial that is already established in many fields; however, the research into their applications in antiviral therapy and the diagnosis of viral diseases is still in its early stage. In this review, we have covered basic physico-chemical characteristics of CDs and the up-to-date knowledge with respect to their antiviral activity and use as platforms for virus detection.

Fluorescent CDs offer many advantages for virus detection adopting either optical or electrochemical sensing approaches. They are characterized by adjustable elemental composition and optical properties that can be tuned by doping or functionalization, and can serve as both electron donors and acceptors. The integration of CDs into biosensing platforms can also lead to the enhancement in sensitivity and specificity when detecting viruses through the presence of their genetic material. A convenient combination of CDs with other nanomaterials (e.g*.*, silver nanoparticles or gold nanobundles) can further broaden the range of potential virus biosensors. Moreover, due to their photoluminescence, CDs can be used for real-time monitoring of subcellular morphological changes upon viral infections, which leads to the future challenge where CDs could serve as a fluorescence probe suitable for virus imaging in living systems. This could be beneficial not only in diagnostics but also in studying the behavior of viruses during the infection. This approach has so far only been explored using semiconductor quantum dots [54], but their toxicity and heavy metal content is a major concern. In the case of CDs, both of these problems are eliminated and they could be potentially the most suitable option for real-time imaging of viruses in living organisms due to their many excellent properties.

Considering the therapy of viral diseases, CDs displayed promising antiviral properties supported by their excellent biocompatibility. Undoubtedly, CDs offer a great opportunity to develop brand new antiviral agents with potentially broad-spectrum activity while keeping their syntheses easy and relatively cheap, depending on the selected precursor. The bottom-up approaches to CD syntheses based on the carbonization of various precursors already possessing antiviral activity seem very promising. A particularly interesting option is the preparation of CDs by cheap green syntheses from natural precursors such as glycyrrhizic acid, curcumin, and carrageenan. It is great news that such simply prepared carbon dots display very promising antiviral properties. Another way to induce or improve the antiviral activity of carbon dots is by post-synthetic modifications with molecules having antiviral effects. Unfortunately, current knowledge of the antiviral activity of carbon dots is based almost exclusively on in vitro models, and to move to in vivo models, we need the CDs to be not only non-toxic and effective in suppressing viral infection, but also to possess hydrophilic properties and perfect colloidal stability under physiological conditions. The combination of all these factors will be a challenge for further research. At the same time, it should be added that it is not easy to obtain reliable in vivo models of viral diseases, and testing the antiviral activity of CDs in vivo will continue to be a challenging discipline. Moreover, there is still a strong need for a deeper understanding of the mechanism of a CD’s antiviral action as well as elucidating the structure–activity relationship in terms of the size, shape, surface charge, etc. Based on most studies, the mechanisms of action consist predominantly in blocking the interaction of the viral particle with the target cell; therefore, the highest antiviral effect is observed mainly during the early stage of the infection. Finally, in order to move towards real medical applications, it is absolutely necessary to support current results with extensive in vivo studies, which remain insufficient at this point.

## Abbreviations

AB: Aminophenylboronic acid
AgNPs: Silver nanoparticles
ALVs-J: Avian leucosis virus subgroup J
AuNPs: Gold nanoparticles
BZM: Benzoxazine monomer
CBBA: 4-carboxy-3-chlorobenzeneboronic acid
CCM: Curcumin
CDs: Carbon dots
CSNs: Carbon dot/silicon dioxide nanospheres
CV: Cyclic voltammetry
DPV: Differential pulse voltammetry
dsDNA: Double-stranded deoxyribonucleic acid
dsRNA: Double-stranded ribonucleic acid
EDA: 2,2′-(ethylenedioxy)bis(ethylamine)
EDC: 1-Ethyl-3-(3-dimethylaminopropyl)carbodiimide
EIS: Electrochemical impedance spectroscopy
ELISA: Enzyme-linked immunosorbent assay
EPA: 3-ethoxypropylamine
EV71: Enterovirus 71
FAdVs: Fowl adenovirus
FCS: Fluorescent carbon dot/silicon dioxide
GCE: Glassy carbon electrode
GQDs: Graphene quantum dots
HBGA: Histo-blood group antigen
HBsAg: Hepatitis B surface antigen
HCoV-229E: Human coronavirus 229E
HCV: Hepatitis C virus
HEV: Hepatitis E virus
HIV-1: Human immunodeficiency virus 1
HSV-1: Herpes simplex virus type 1
IFN: Interferon
ISGs: Interferon-stimulated genes
JEV: Japanese encephalitis virus
KSHV: Kaposi’s sarcoma-associated herpesvirus
LNA: Locked nucleic acid
LOD: Limit of detection
MERS-CoV: Middle East respiratory syndrome-related coronavirus
miRNA: Microribonucleic acid
mRNA: Messenger ribonucleic acid
MS2: Bacteriophage MS2
N,S-GQDs: Nitrogen- and thiol-doped graphene quantum dots
NB: Nanobundle
NHS: N-hydroxysuccinimide
NIR: Near-infrared
NoV: Norovirus
PANI: Polyaniline
PDA: Polydopamine
pDNA: Probe deoxyribonucleic acid
PEC: Photoelectrochemical
PEDV: Porcine epidemic diarrhea virus
PEG: Polyethylene glycol
PEI: Polyethyleneimine
PEL: Primary effusion lymphoma
pfu: Plaque-forming unit
PL: Photoluminescence
PLL: Poly-L-lysine
PRRSV: Porcine reproductive and respiratory syndrome virus
PRV: Pseudorabies virus
PS: Polymer nanosphere
QY: Quantum yield
ROS: Reactive oxygen species
RT-qPCR: Reverse transcription quantitative real-time polymerase chain reaction
SFTSV: Severe fever with thrombocytopenia syndrome virus
ssDNA: Single-stranded deoxyribonucleic acid
ssRNA: Single-stranded ribonucleic acid
TCID_50_: Median tissue culture infectious dose
UV: Ultra-violet
VLPs: Virus-like particles
WSSV: White spot syndrome virus
